# Supplementary material for: In-situ electrochemical reconstruction and modulation of adsorbed hydrogen coverage in cobalt/ruthenium-based catalyst boost electroreduction of nitrate to ammonia
Source: Nat Commun. 2024 Oct 3;15:8583. doi: 10.1038/s41467-024-52780-x (PMC11450097; doi:10.1038/s41467-024-52780-x)
Supplement: Supplementary file 1 — Supplementary Information [file 41467_2024_52780_MOESM1_ESM.pdf]

In-situ Electrochemical Reconstruction and Modulation of Adsorbed Hydrogen Coverage in Cobalt/Ruthenium-based Catalyst Boost Electroreduction of Nitrate to Ammonia

Jian Zhang[1], Thomas Quast[1], Bashir Eid[1], Yen-Ting Chen[2], Ridha Zerdoumi[1], Stefan Dieckhöfer[1], João R. C. Junqueira[1], Sabine Seisel[1], Wolfgang Schuhmann[1]*

[1]Analytical Chemistry - Center for Electrochemical Sciences (CES); Faculty of Chemistry and Biochemistry, Ruhr University Bochum; Universitätsstr. 150; D-44780 Bochum; Germany.

[2]Center for Solvation Science (ZEMOS), Ruhr University Bochum, Universitätsstr. 150; D-44801 Bochum, Germany

E-mail: wolfgang.schuhmann@rub.de

Supplementary Figures

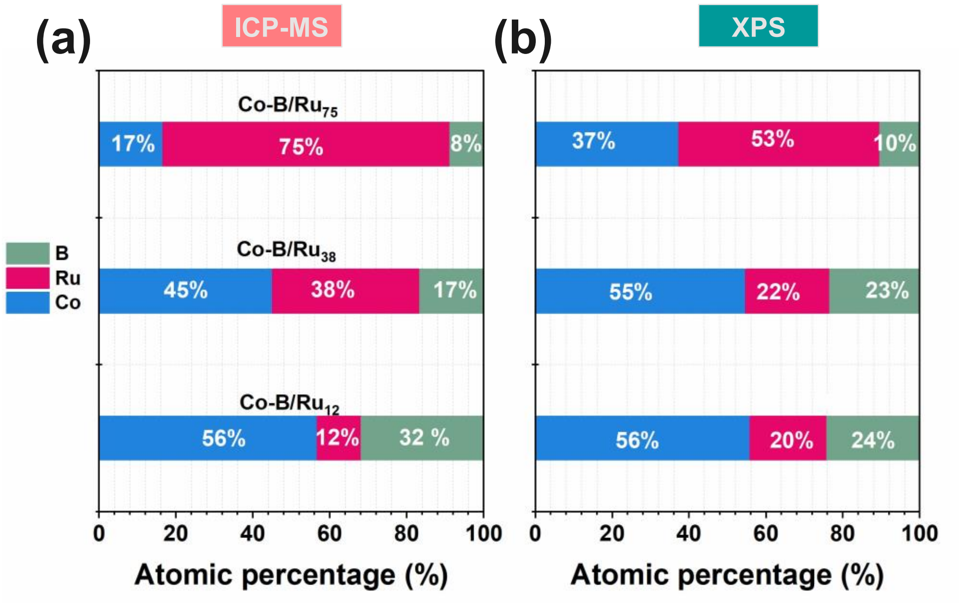

Supplementary Fig. S1 | Elemental compositions of the catalysts. (**a**) ICP-MS-determined atomic percentage of Co, Ru, and B of the synthesized catalysts. (**b**) XPS-determined atomic percentage of Co, Ru, and B at the surface of the synthesized catalysts.

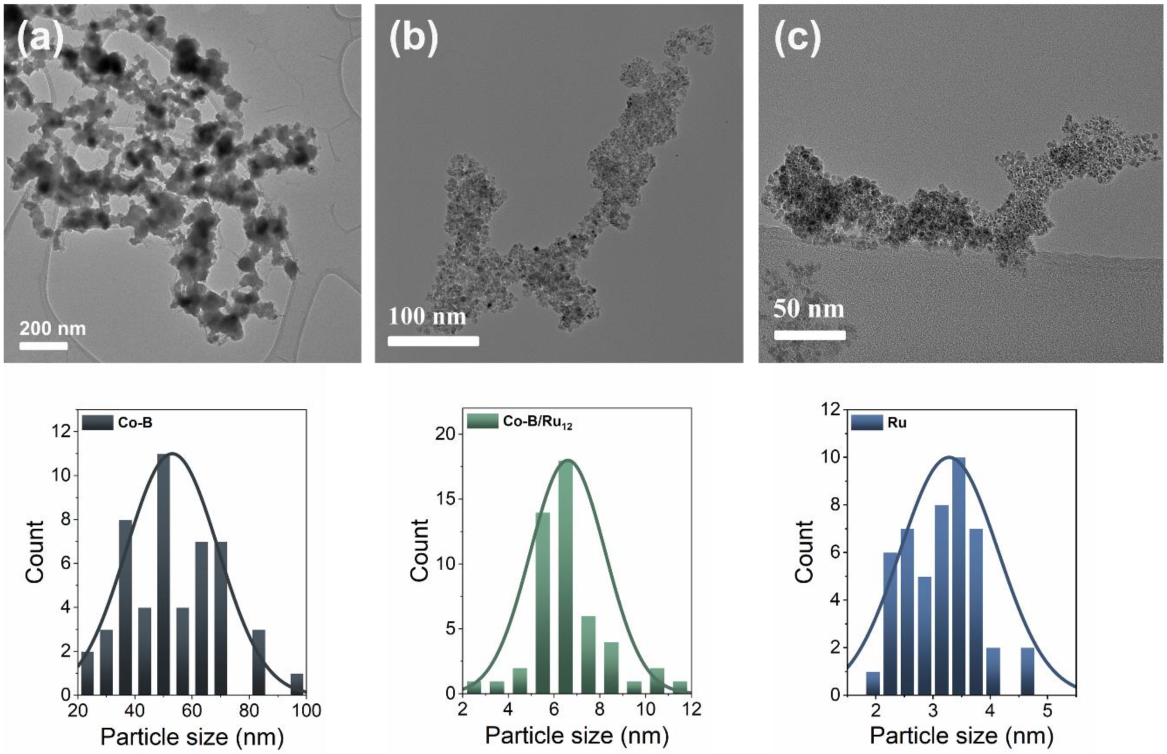

Supplementary Fig. S2 | Particle size distribution of the catalysts. (**a**) TEM images of Co-B and the corresponding size distribution curve. (**b**) TEM images of Co-B/Ru_{12} and the corresponding size distribution curve. (**c**) TEM images of Ru and the corresponding size distribution curve.

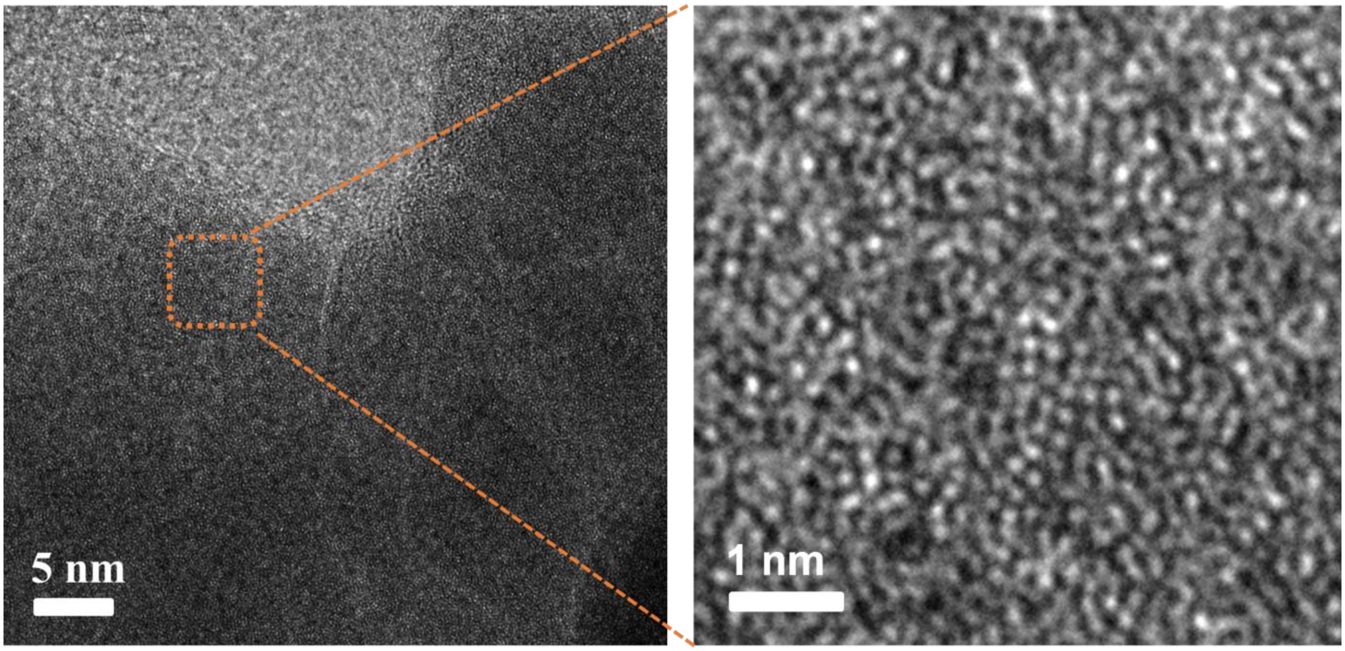

Supplementary Fig. S3 | Phase analysis of Co-B. TEM and the corresponding HR-TEM images of Co-B.

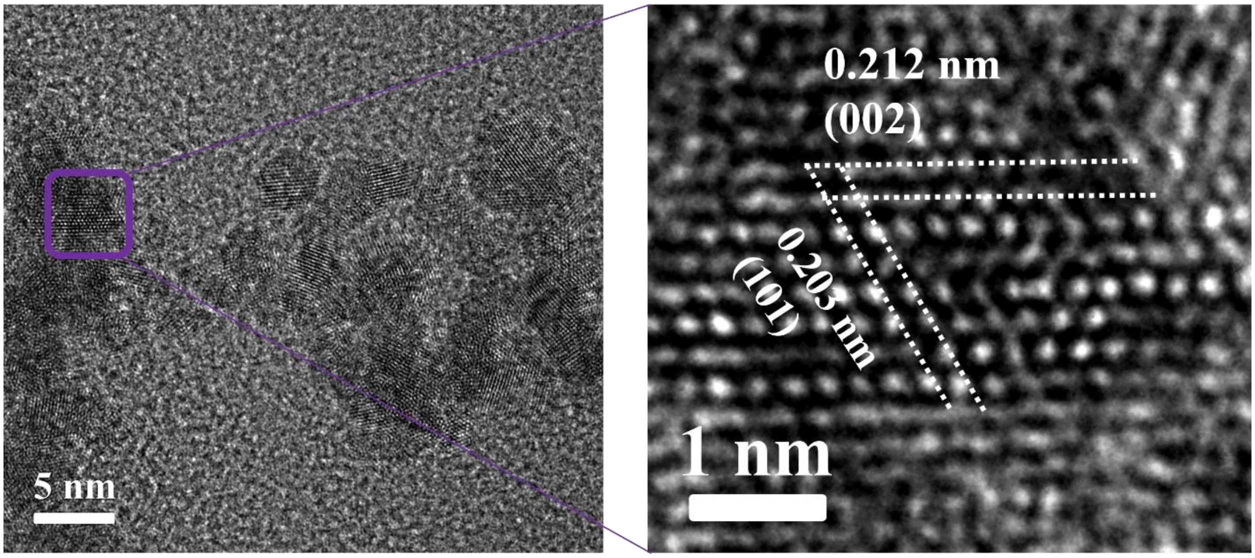

Supplementary Fig. S4 | Phase analysis of Ru. TEM and the corresponding HR-TEM images showing the lattice fringes of hexagonal metallic Ru.

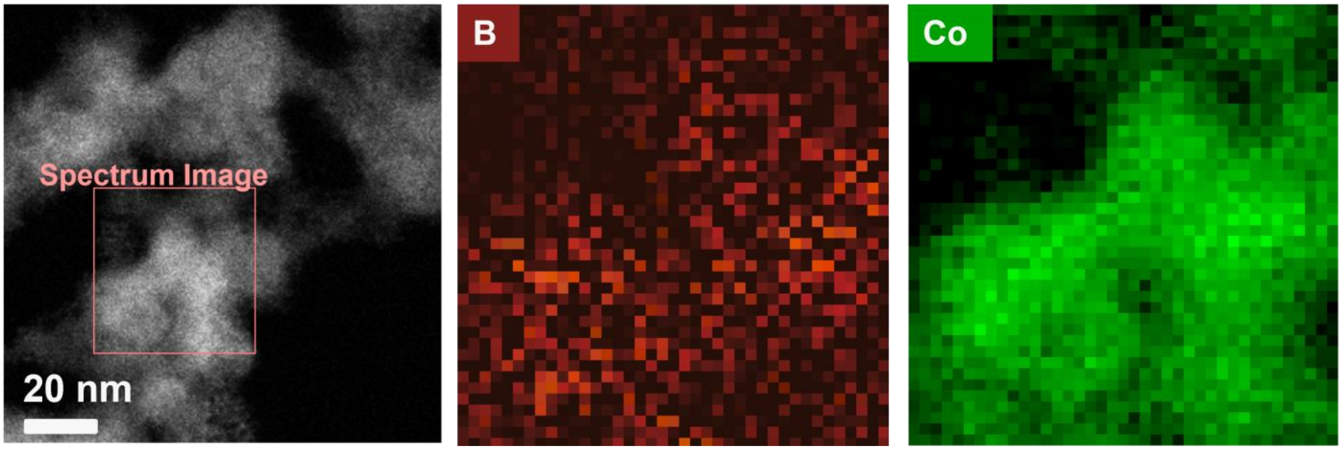

Supplementary Fig. S5 | Elements distribution at nanoscale. STEM and the corresponding EELS mapping images of B and Co.

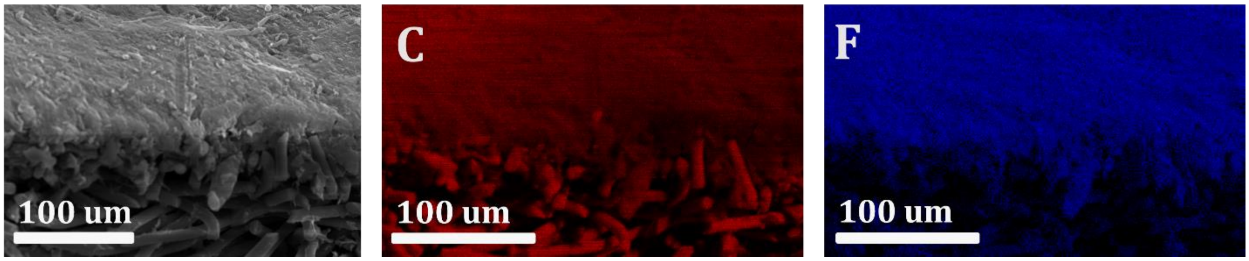

Supplementary Fig. S6 | Element distribution of carbon paper substrate. SEM image and corresponding EDS mapping of C and F (PTFE).

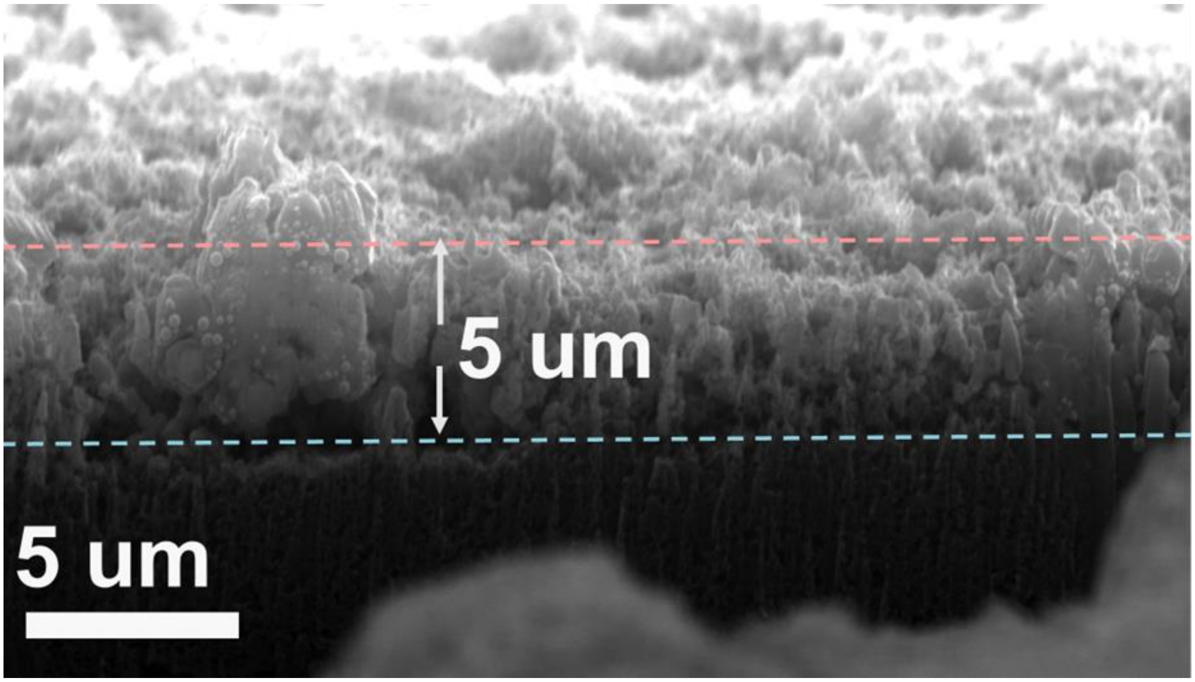

Supplementary Fig. S7 | Cross-section view of catalyst modified working electrode. Focus ion beam-cut SEM image of Co-B/Ru_{12} on carbon paper (captured from a near-perpendicular angle to the electron beam)

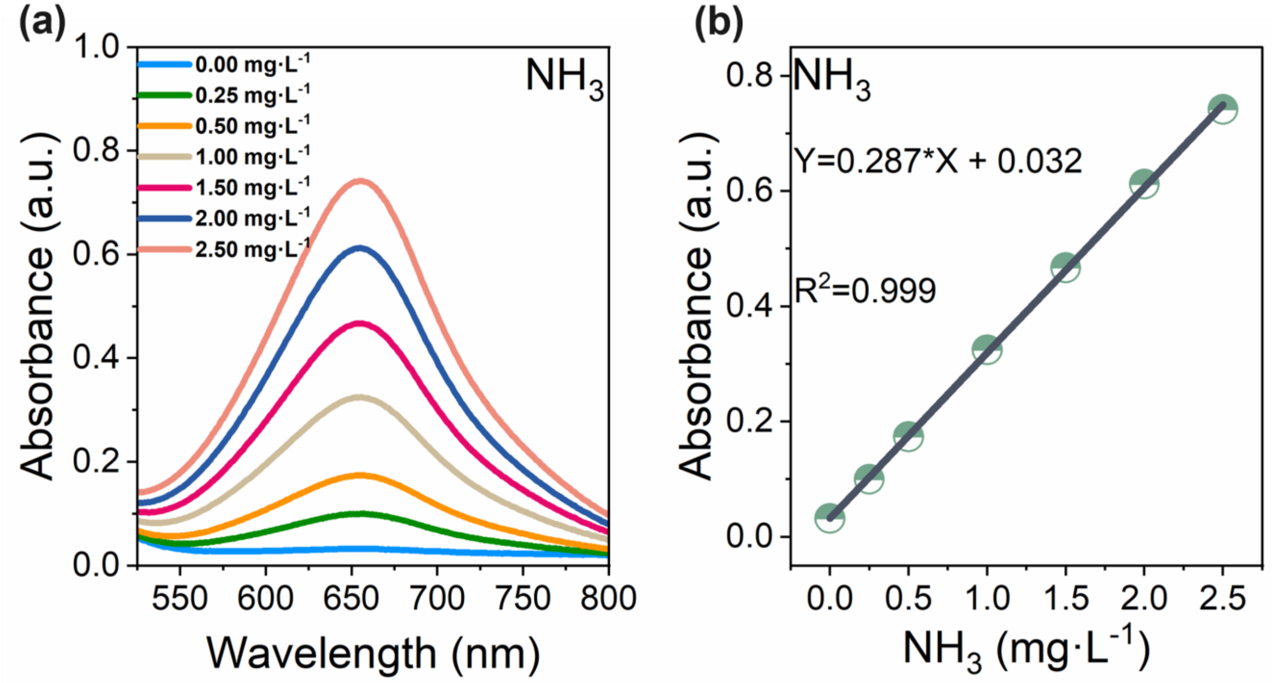

Supplementary Fig. S8 | NH_3 quantification using UV-Vis absorption spectroscopy. UV−Vis absorption spectra (**a**) and corresponding calibration curve (**b**) for the NH_3 assay using the indophenol blue method.

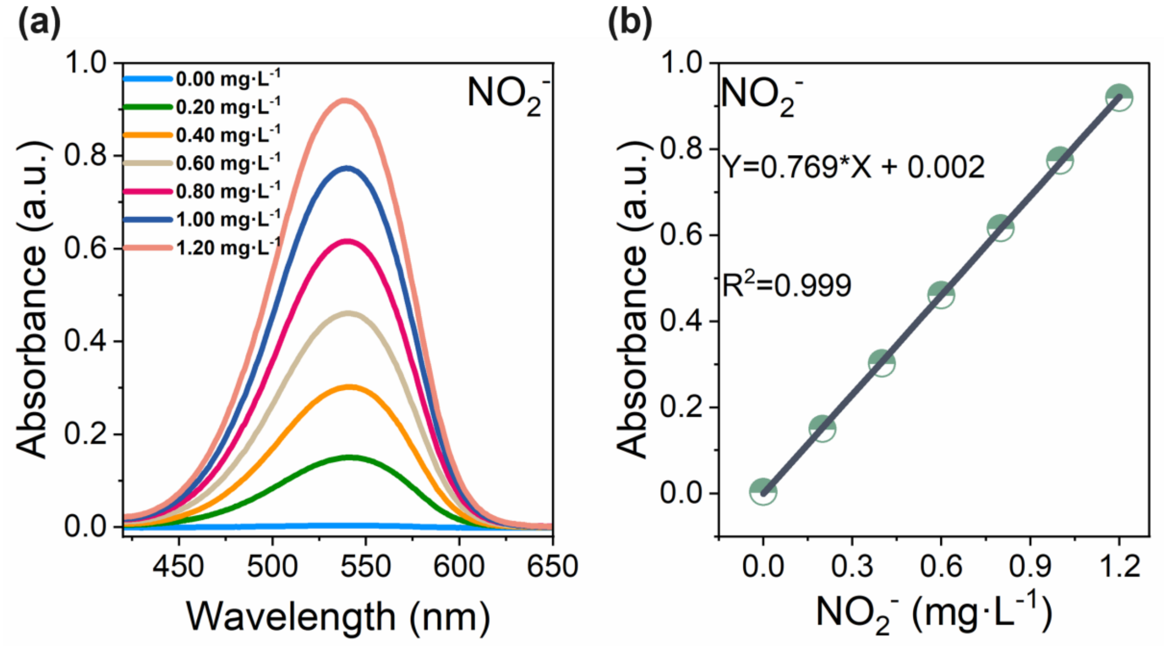

Supplementary Fig. S9 | NO_2^- quantification using UV-Vis absorption spectroscopy. UV−Vis absorption spectra (**a**) and corresponding calibration curve (**b**) for the NO_2^- assay.

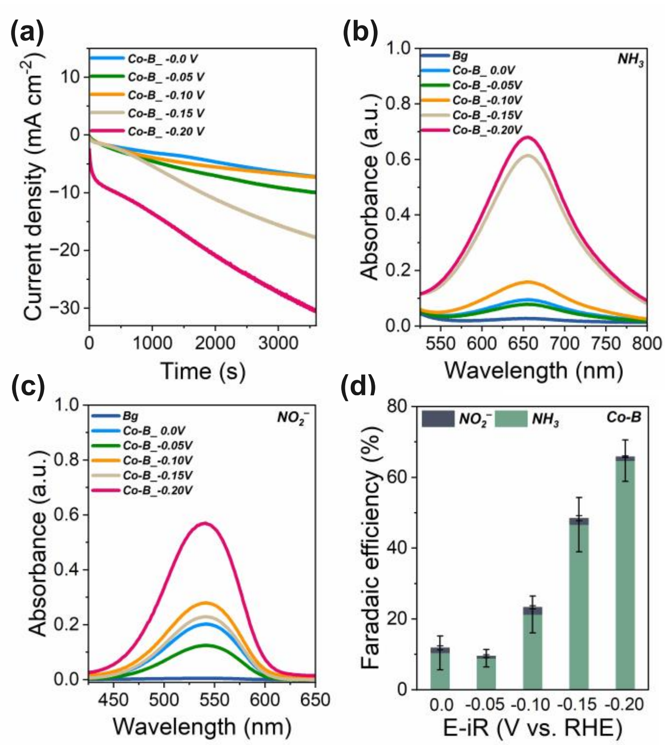

Supplementary Fig. S10 | NH_3 synthesis performance on Co-B at a series of potentials. (**a**) Chronoamperograms of Co-B at different potentials for 1 h in 0.1 mol L^{-1} NaOH and 0.1 mol L^{-1} NO_3^-. UV−Vis absorption spectra for quantification of NH_3 (**b**) and NO_2^- (**c**). Note that for the detection of the NH_4^+, the post−electrolysis electrolytes at 0 V, –0.05 V, −0.1 V, –0.15 V were diluted 2 times, while the one at –0.2 V was diluted 4 times. As for the NO_2^- tests, the post−electrolysis electrolytes at 0 V, –0.05 V, −0.1 V, –0.15 V, −0.2 V were diluted 2.5 times. (**d**) Faradaic efficiency (FE) of NH_3 and NO_2^-. Error bars denote the standard deviations from at least three independent measurements.

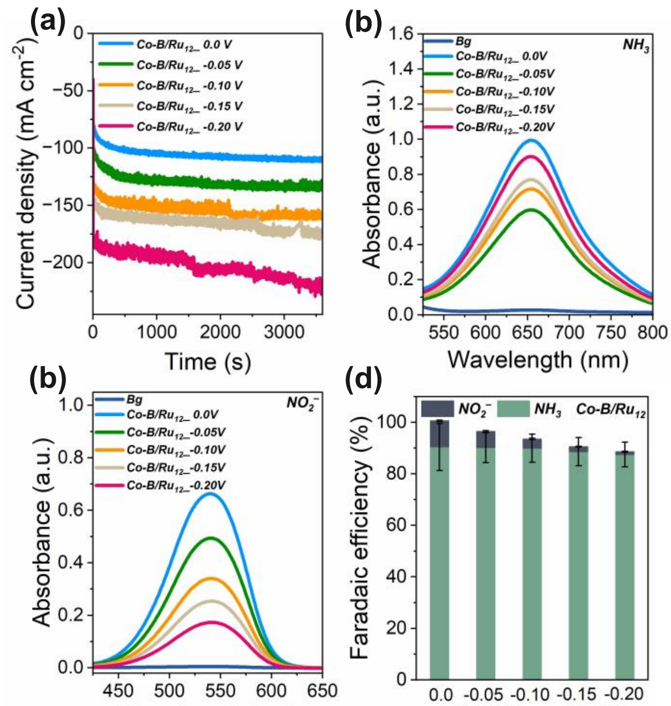

Supplementary Fig. S11 | NH_3 synthesis performance on Co-B/Ru_{12} at a series of potentials. (**a**) Chronoamperograms of Co-B/Ru_{12} at different potentials for 1 h in 0.1 mol L^{-1} NaOH and 0.1 mol L^{-1} NO_3^-. UV−Vis absorption spectra for the quantification of NH_3 (**b**) and NO_2^- (**c**). Note that for the detection of NH_4^+, the post−electrolysis electrolytes at –0.05 V, −0.1 V, –0.15 V, –0.2 V were diluted 40 times, while that at 0 V was diluted 20 times. As for the NO_2^- tests, the post−electrolysis electrolytes at 0 V, –0.05 V, −0.1 V, –0.15 V, −0.2 V were diluted 25 times. (**d**) Faradaic efficiency (FE) of NH_3 and NO_2^-. Error bars denote the standard deviations from at least three independent measurements.

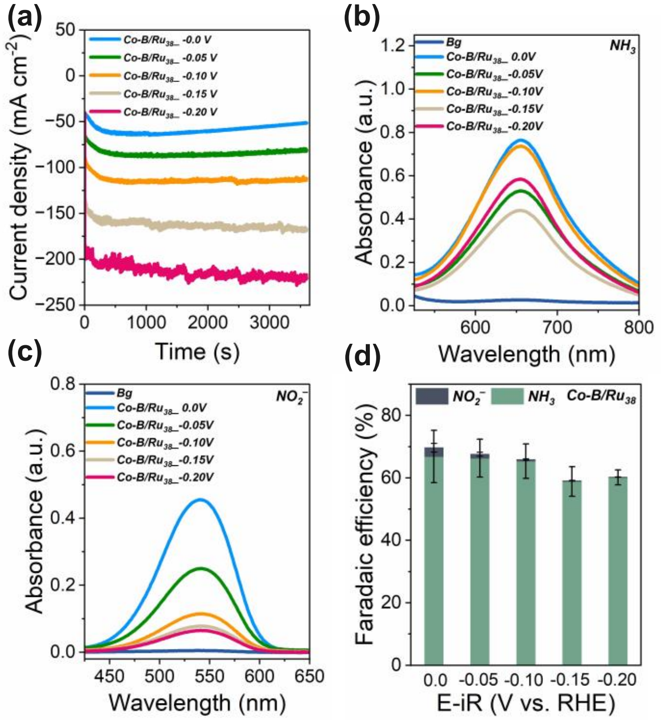

Supplementary Fig. S12 | NH_3 synthesis performance on Co-B/Ru_{38} at a series of potentials. (**a**) Chronoamperograms of Co-B/Ru_{38} at different potentials for 1 h in 0.1 mol L^{-1} NaOH and 0.1 mol L^{-1} NO_3^-. UV−Vis absorption spectra for the quantification of NH_3 (**b**) and NO_2^- (**c**). Note that for the detection of NH_4^+, the post−electrolysis electrolyte at 0 V was diluted 10 times, at –0.05 V, −0.1 V were diluted 20 times, while that at –0.15 V, −0.2 V were diluted 40 times. As for the NO_2^- tests, the post−electrolysis electrolytes at 0 V, –0.05 V, −0.1 V, –0.15 V, −0.2 V were diluted 25 times. (**d**) Faradaic efficiency (FE) of NH_3 and NO_2^-. Error bars denote the standard deviations from at least three independent measurements.

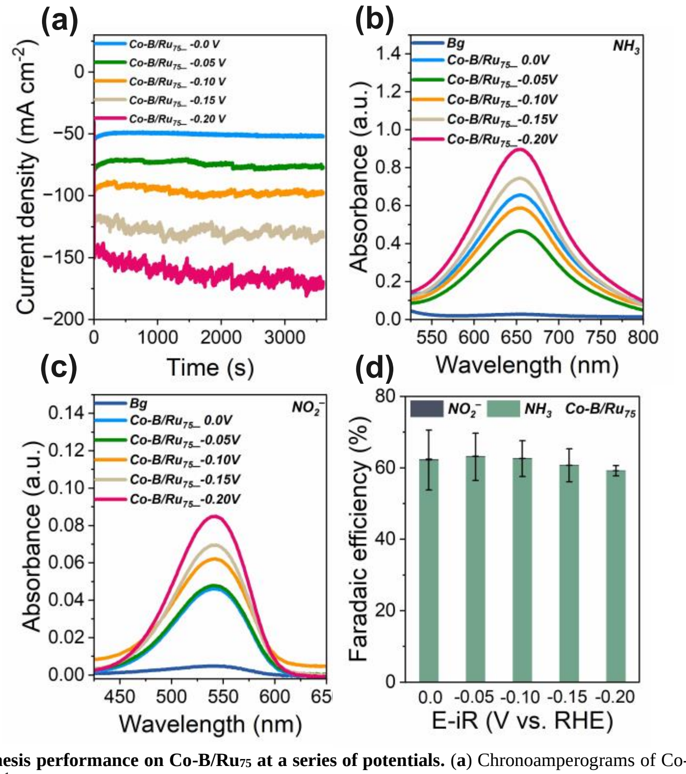

Supplementary Fig. S13 | NH_3 synthesis performance on Co-B/Ru_{75} at a series of potentials. (**a**) Chronoamperograms of Co-B/Ru_{75} at different potentials for 1 h in 0.1 mol L^{-1} NaOH and 0.1 mol L^{-1} NO_3^-. UV−Vis absorption spectra for the quantification of NH_3 (**b**) and NO_2^- (**c**). Note that for the detection of NH_4^+, the post−electrolysis electrolytes at –0.05 V,−0.1 V, –0.15 V, −0.2 V were diluted 20 times, , while that at 0 V were diluted 10 times. As for the NO_2^- tests, the

post−electrolysis electrolytes at 0 V, –0.05 V,−0.1 V, –0.15 V, −0.2 V were diluted 5 times. (**d**) Faradaic efficiency (FE) of NH_3 and NO_2^-. Error bars denote the standard deviations from at least three independent measurements.

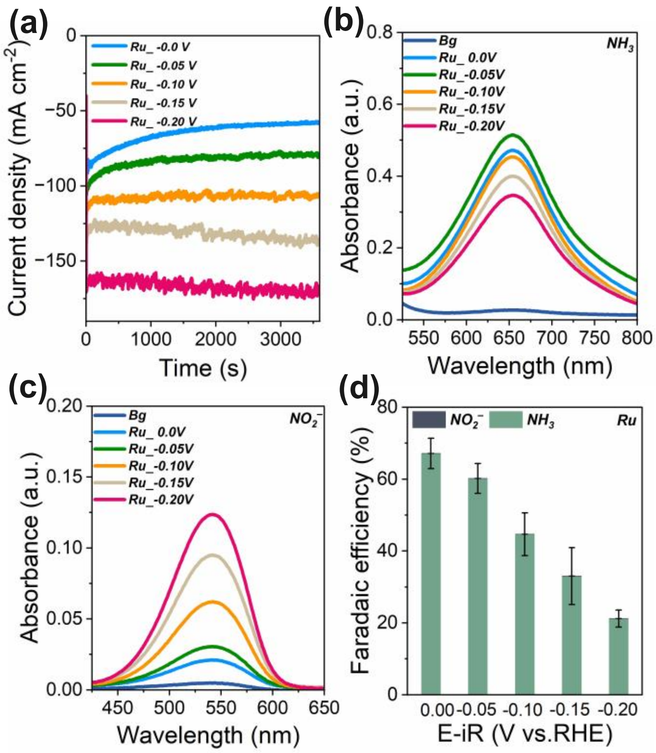

Supplementary Fig. S14 | NH_3 synthesis performance on Ru at a series of potentials. (**a**) Chronoamperograms of Ru at different potentials for 1 h in 0.1 mol L^{-1} NaOH and 0.1 mol L^{-1} NO_3^-. UV−Vis absorption spectra for the quantification of NH_3 (**b**) and NO_2^- (**c**). Note that for the detection of the NH_4^+, the post−electrolysis electrolytes at 0 V, –0.05 V, −0.1 V, –0.15 V, −0.2 V were diluted 20 times. As for the NO_2^- tests, the post−electrolysis electrolytes at 0 V, –0.05 V,−0.1 V, –0.15 V, −0.2 V were diluted 2.5 times. (**d**) Faradaic efficiency (FE) of NH_3 and NO_2^-. Error bars denote the standard deviations from at least three independent measurements.

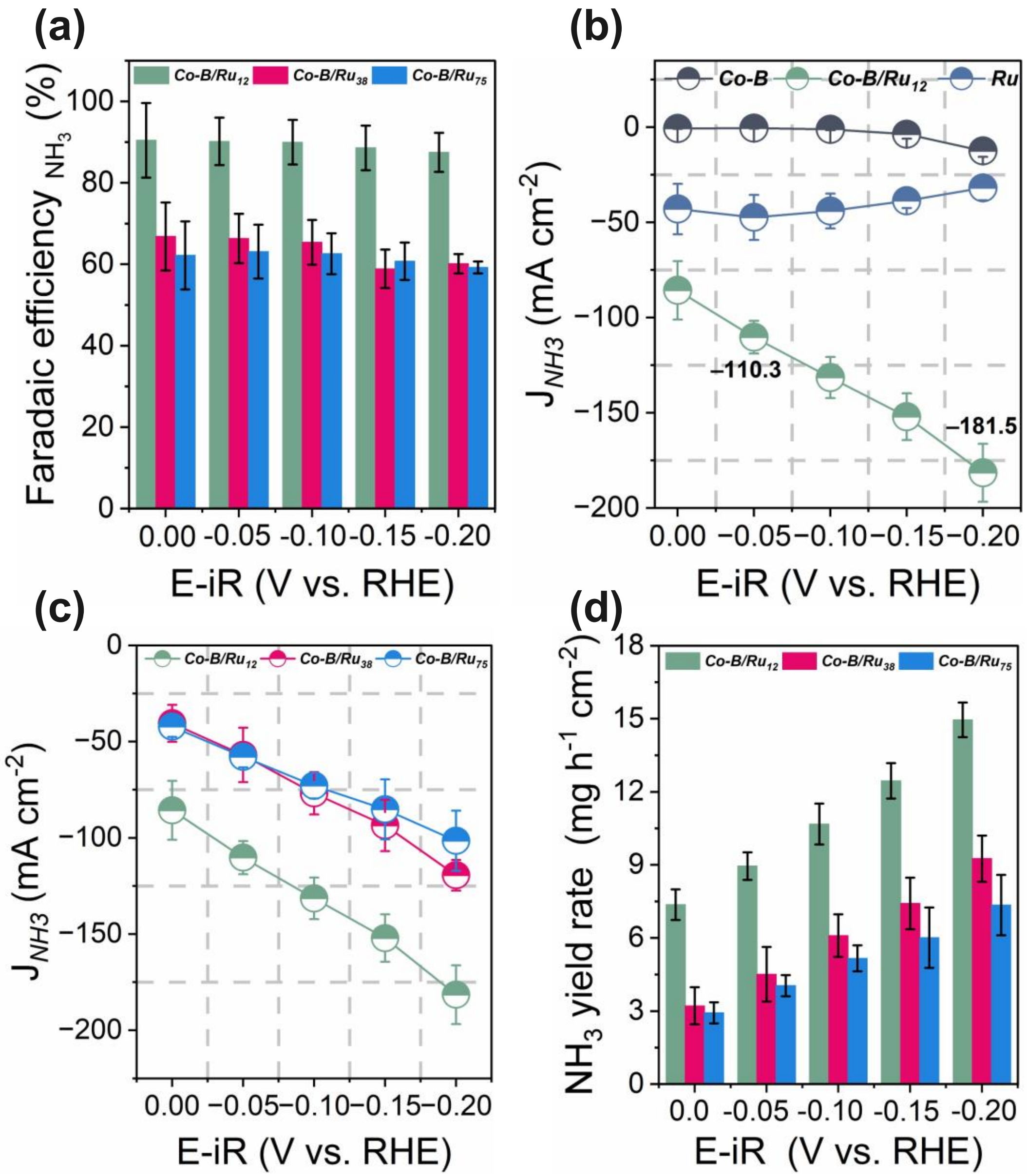

Supplementary Fig. S15 | Comparison of NO_3RR performance of various catalysts. Faradaic efficiencies (**a**), partial current densities of NH_3 (**c**), yield rate for NH_3 (**d**) on Co-B/Ru_{12}, Co-B/Ru_{38}, Co-B/Ru_{75}. Partial current densities of NH_3 on Co-B/Ru_{12}, Co-B, Ru (**b**). Error bars denote the standard deviations from at least three independent measurements.

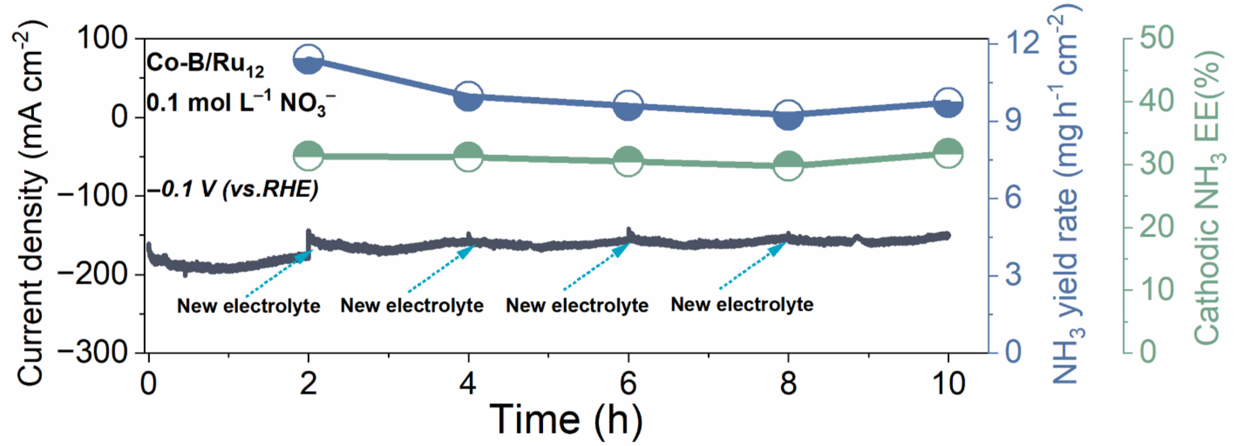

Supplementary Fig. S16 | Stability test on Co-B/Ru_{12}. Chronoamperometric stability test of Co-B/Ru_{12} at –0.1 V (vs RHE) in 0.1 mol L^{-1} NO_3^-, corresponding yield rate for NH_3, and cathodic energy efficiencies for NH_3.

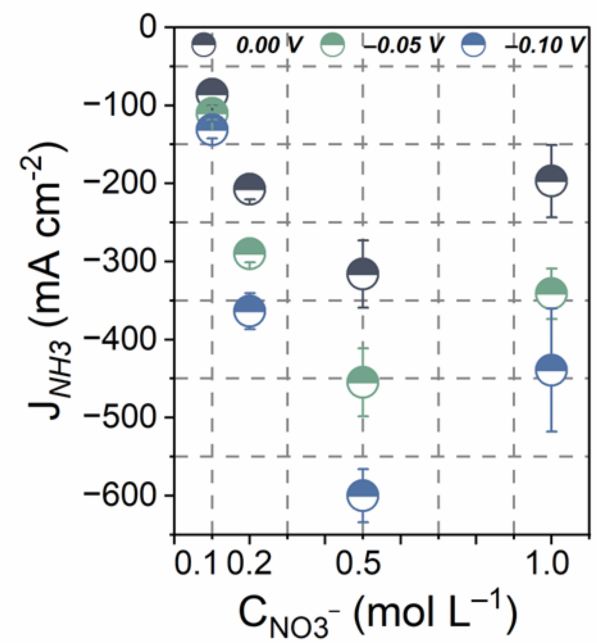

Supplementary Fig. S17 | Partial current density of NH_3 (J_{NH3}) comparison. J_{NH3} of Co-B/Ru_{12} in different concentration of NO_3^- in potential range from 0V- -0.1 V (Vs. RHE). Error bars denote the standard deviations from at least three independent measurements.

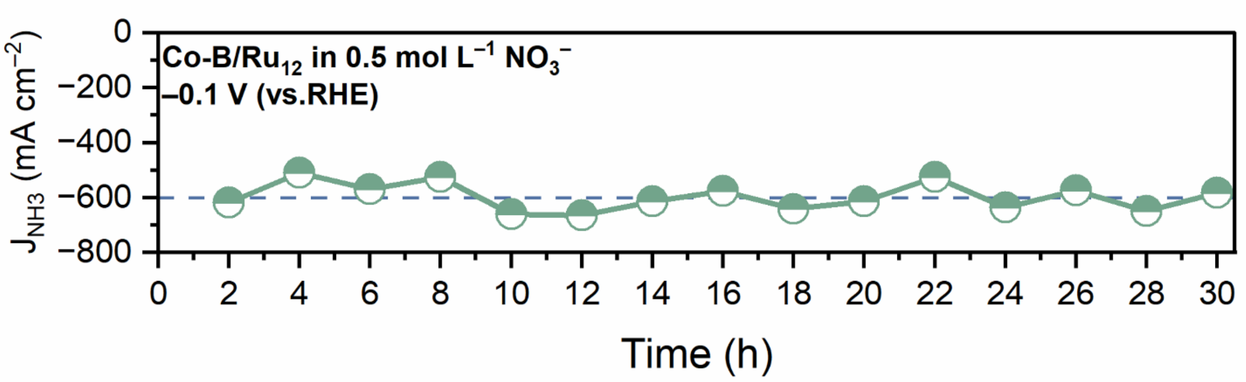

Supplementary Fig. S18 | Stability test on Co-B/Ru_{12}. Chronoamperometric stability test of Co-B/Ru_{12} at –0.1 V (vs RHE) in 0.1 mol L^{-1} NO_3^-, showing the partial current for NH_3 (with electrolyte replacement each 2 h).

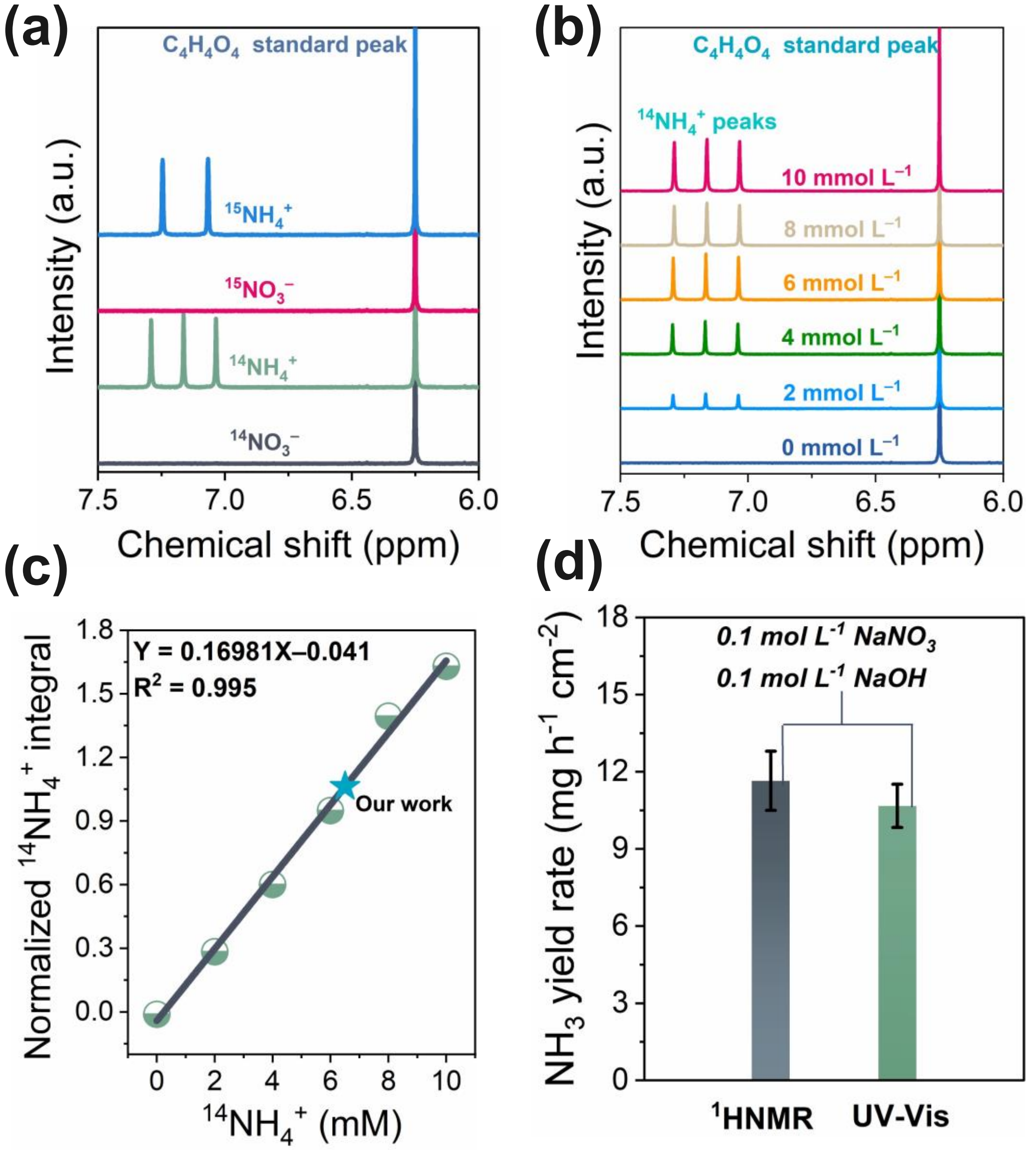

Supplementary Fig. S19 | $^{15}NH_4^+$ detection and $^{14}NH_4^+$ quantification by ^{1}H NMR spectroscopy. (**a**) ^{1}H NMR spectra of the electrolytes after electrocatalysis using 0.1 mol L^{-1} $^{15}NO_3^-$ or 0.1 mol L^{-1} $^{14}NO_3^-$ in 0.1 mol L^{-1} NaOH as nitrogen source. ^{1}H NMR of the fresh electrolytes before electrolysis (marked as $^{15}NO_3^-$ and $^{14}NO_3^-$) are provided as controls showing no background signals of ammonia. (**b**) ^{1}H NMR spectra of different concentrations $^{14}NH_4^+$. A constant concentration of maleic acid was used as an internal standard with a proton signal at d = 6.25 ppm. (**c**) Calibration curve for $^{14}NH_4^+$ detection using ^{1}H NMR, where the $^{14}NH_4^+$ peak area integrals were normalized by that of maleic acid. The normalized peak area integral of $^{14}NH_4^+$ is positively correlated to the concentrations of $^{14}NH_4^+$ [$^{14}NH_4^+$]. (**d**) Comparison of the ammonia yield rate over Co quantified by UV−Vis spectra and ^{1}H NMR. The electrolysis was carried out at −0.1 V (vs. RHE) for 1 h in 0.1 mol L^{-1} NaOH with 0.1 mol L^{-1} $^{14}NO_3^-$. Error bars denote the standard deviations from at least three independent measurements.

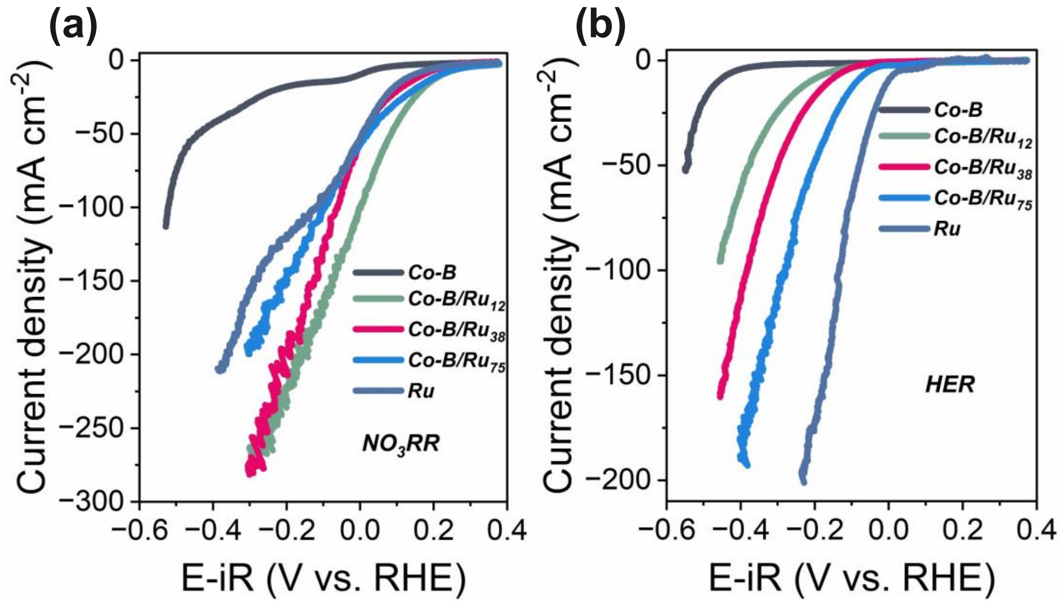

Supplementary Fig. S20 | Comparison of NO₃RR and HER on various catalysts. LSV curves of various catalyst in 0.1 mol L^{-1} NaOH with 0.1 mol L^{-1} NO_3^- (**a**) and in in 0.1 mol L^{-1} NaOH (**b**).

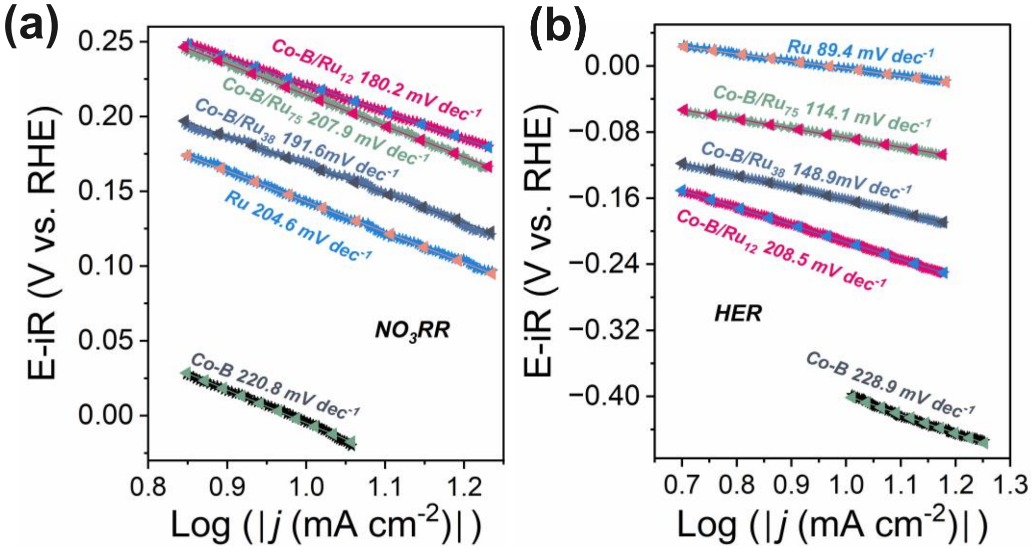

Supplementary Fig. S21 | NO₃RR and HER reaction kinetics comparison on various catalyst. Tafel slopes of various catalyst in 0.1 mol L^{-1} NaOH with 0.1 mol L^{-1} NO_3^- (**a**) and in 0.1 mol L^{-1} NaOH (**b**).

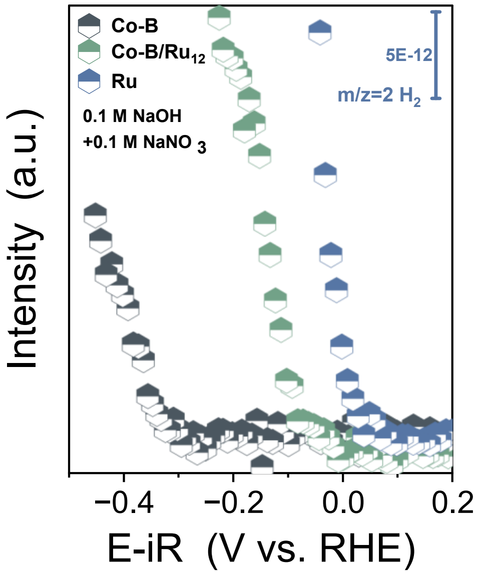

Supplementary Fig. S22 | DEMS analysis. Plots showing the ion current (H_2) versus potential obtained using DEMS. The ion current is not corrected/deconvoluted.

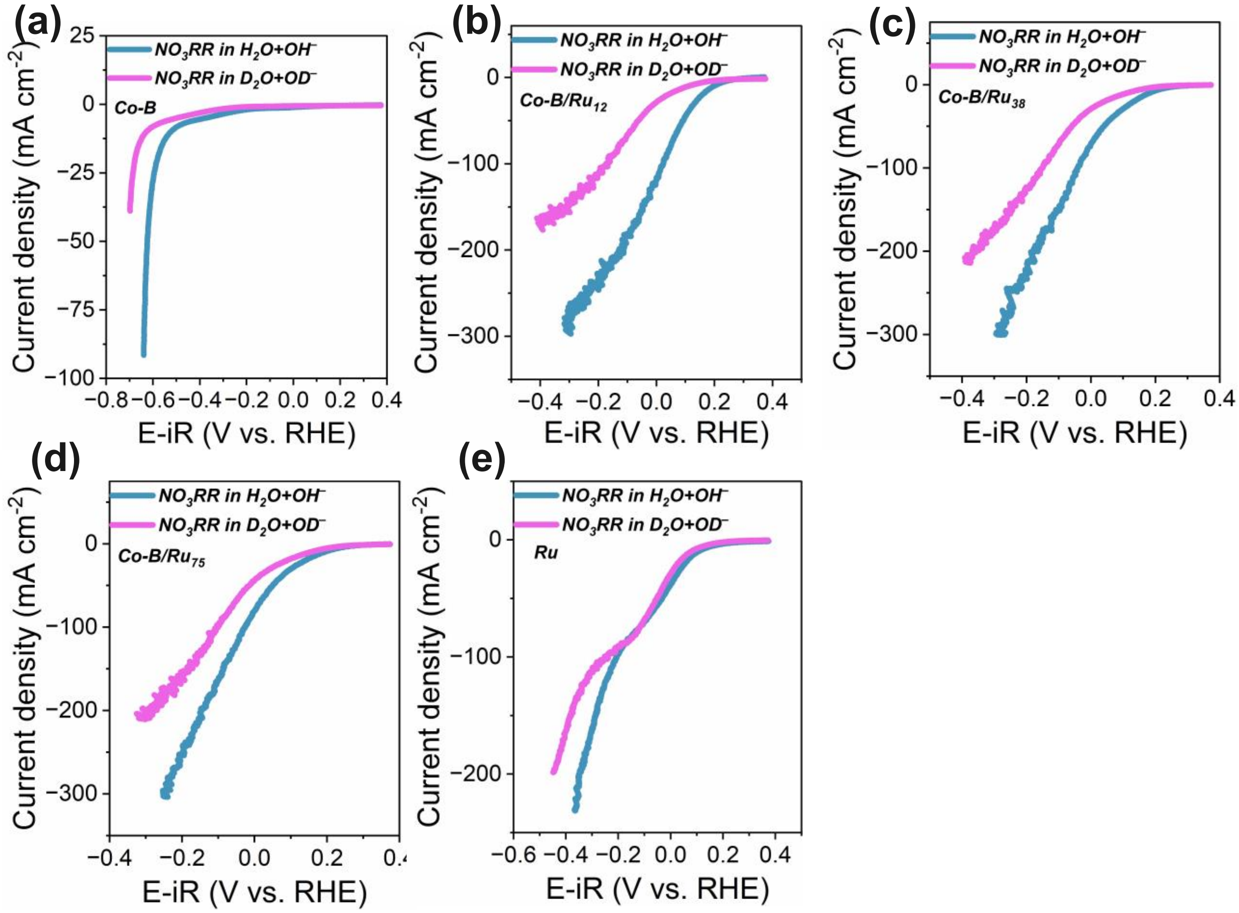

Supplementary Fig. S23 | KIE measurements on various catalysts. LSV curves of Co-B (**a**), Co-B/Ru_{12} (**b**), Co-B/Ru38 (**c**), Co-B/Ru75 (**d**), Ru (**e**) in an electrolyte of 0.1 mol L^{-1} NaOD with 0.1 mol L^{-1} NO_3^- in D_2O and in an electrolyte of 0.1 mol L^{-1} NaOH with 0.1 mol L^{-1} NO_3^- in H_2O for the determination of the kinetic isotope effect (KIE) values.

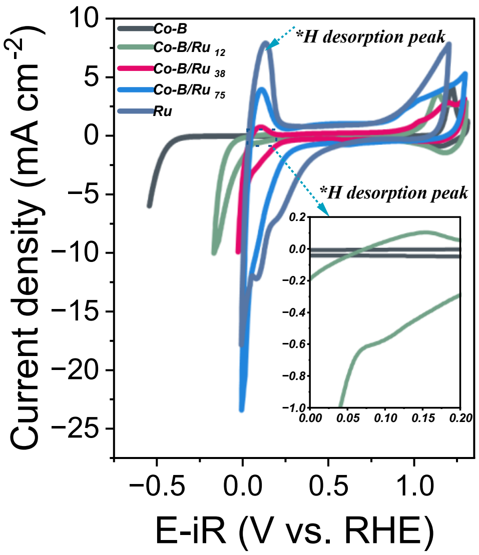

Supplementary Fig. S24 | Adsorbed hydrogen (*H) analysis. Cyclic voltammograms (CV) for the determination of the *H coverage on different catalysts in Ar−saturated 0.1 M NaOH.

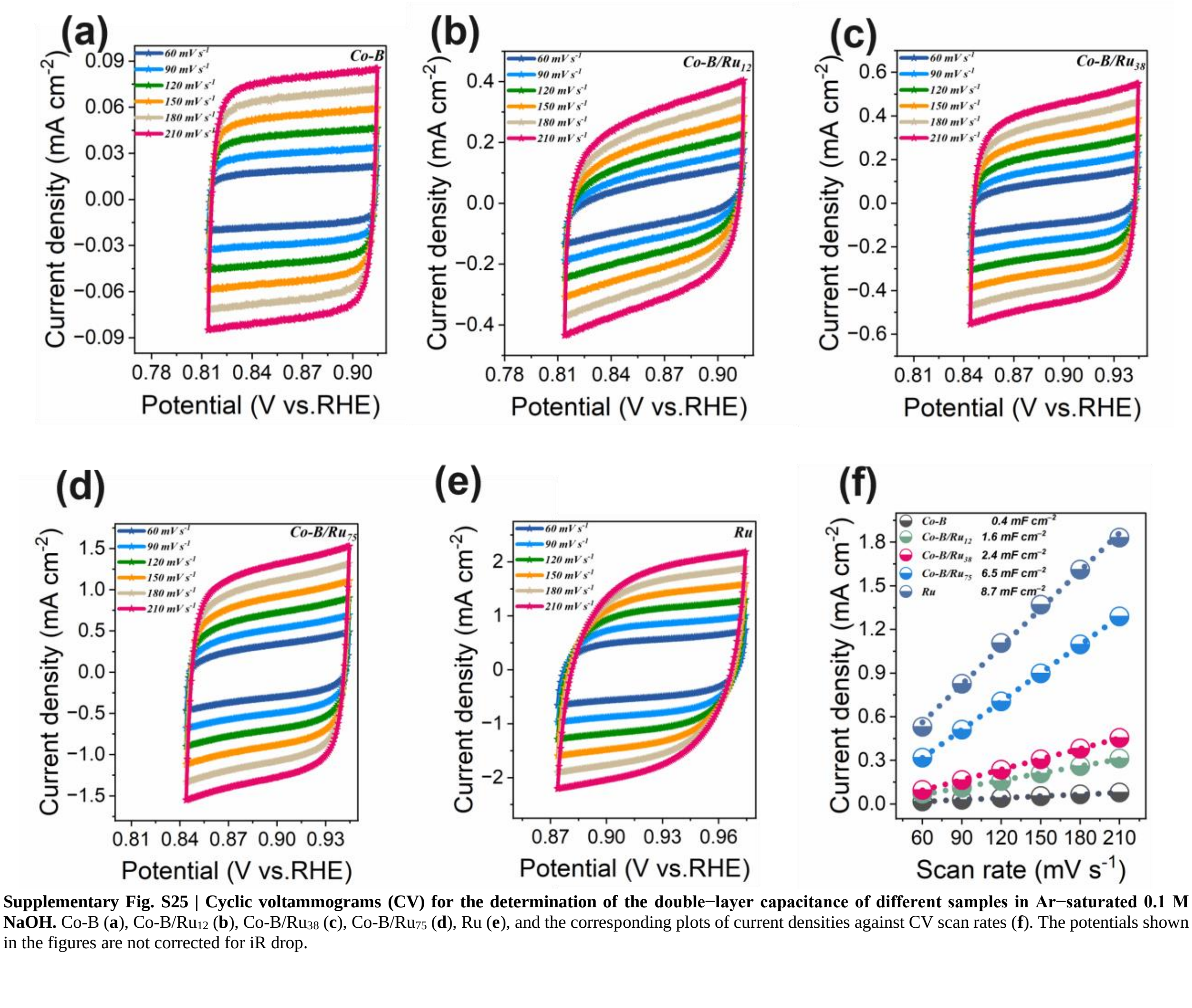

Supplementary Fig. S25 | Cyclic voltammograms (CV) for the determination of the double−layer capacitance of different samples in Ar−saturated 0.1 M NaOH. Co-B (**a**), Co-B/Ru_{12} (**b**), Co-B/Ru_{38} (**c**), Co-B/Ru_{75} (**d**), Ru (**e**), and the corresponding plots of current densities against CV scan rates (**f**). The potentials shown in the figures are not corrected for iR drop.

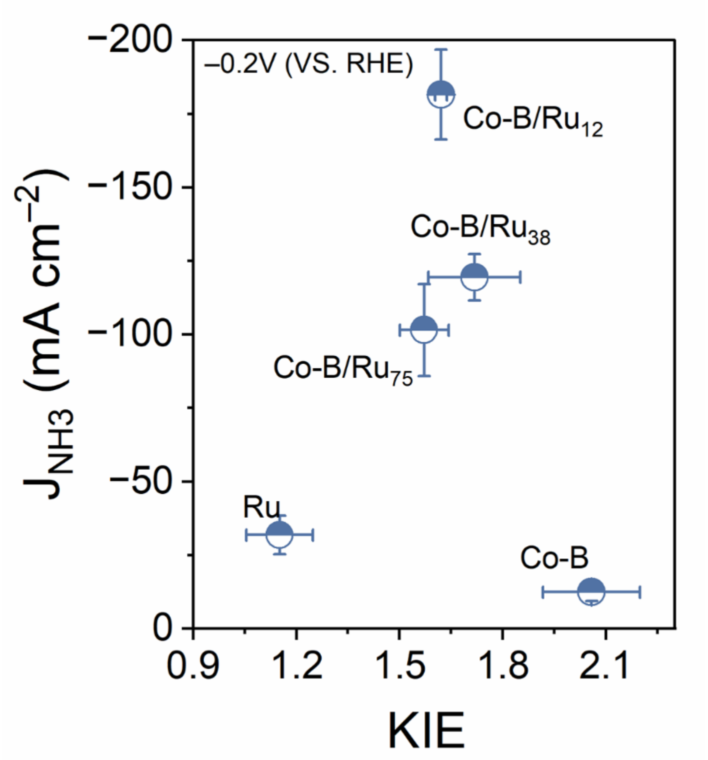

Supplementary Fig. S26 | Plot of partial current for NH_3 against the KIE values of different catalysts. Error bars denote the standard deviations from at least three independent measurements.

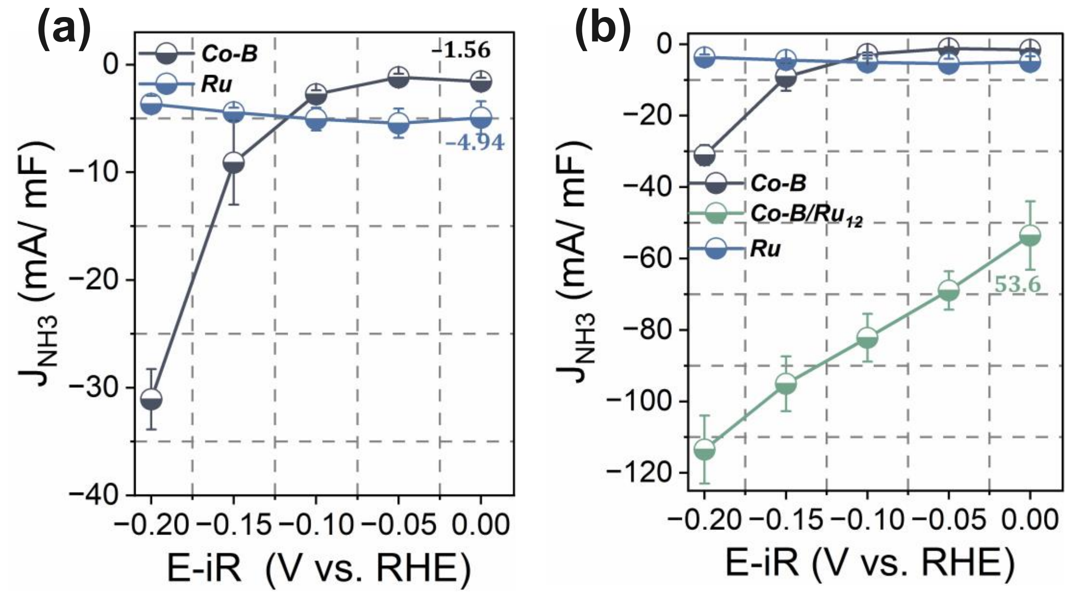

Supplementary Fig. S27 | Comparison of partial current for NH_3 normalized by C_{dl} on various catalysts. (a) partial current for NH_3 normalized by C_{dl} on Co-B and Ru. (b) partial current for NH_3 normalized by C_{dl} on Co-B and Ru. Error bars denote the standard deviations from at least three independent measurements.

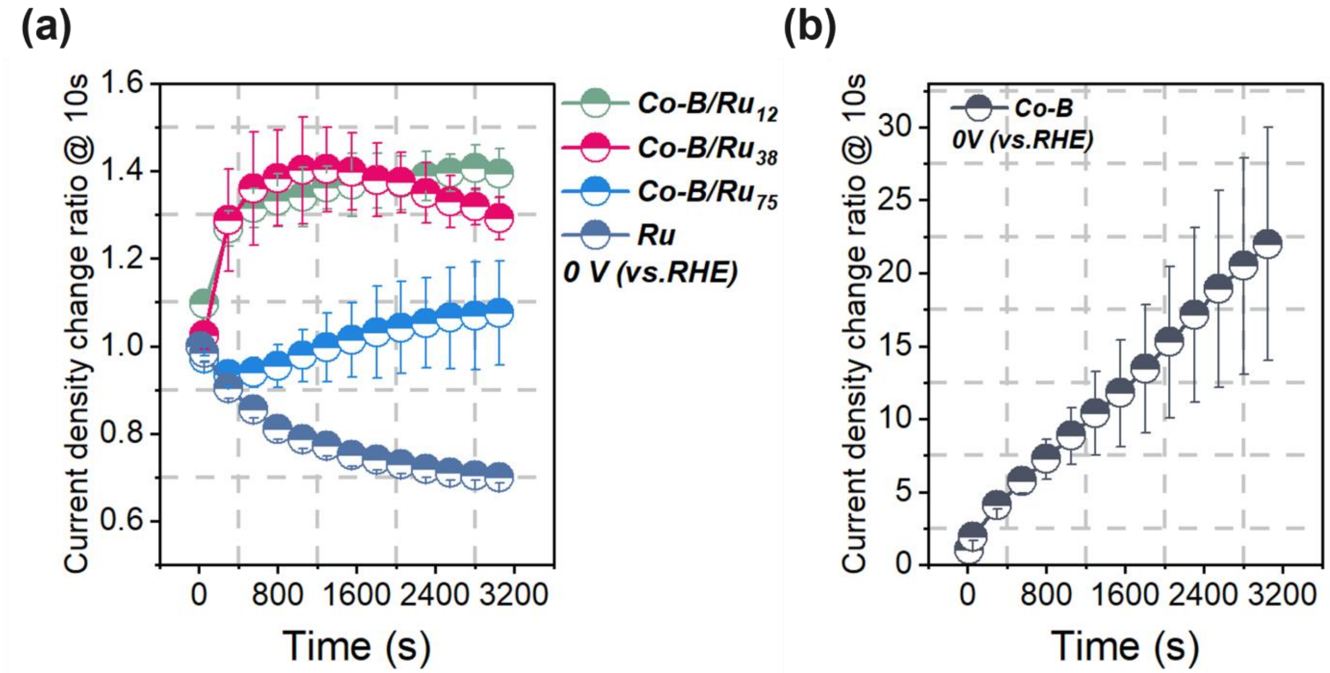

Supplementary Fig. S28 | Current density changes during chronoamperometry measurements. Plots showing the current density change ratio with respect to the value at 10 s of Co-B/Ru_x (**a**) and other catalysts (**b**) at a potential of 0 V (vs. RHE). Error bars denote the standard deviations from at least three independent measurements.

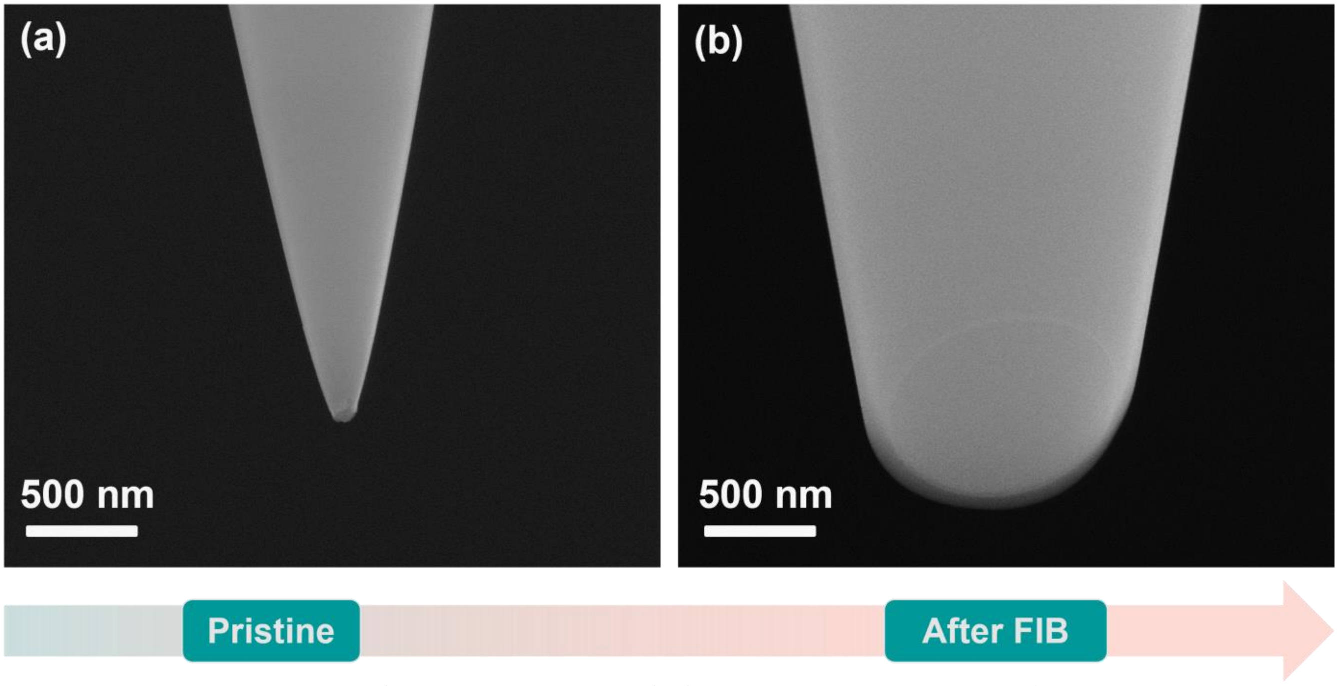

Supplementary Fig. S29 | Morphology changes of a carbon nanoelectrode (CNE) after focus ion beam (FIB) milling. SEM images of carbon nanoelectrodes before (**a**) and after (**b**) the focus ion beam (FIB) milling process.

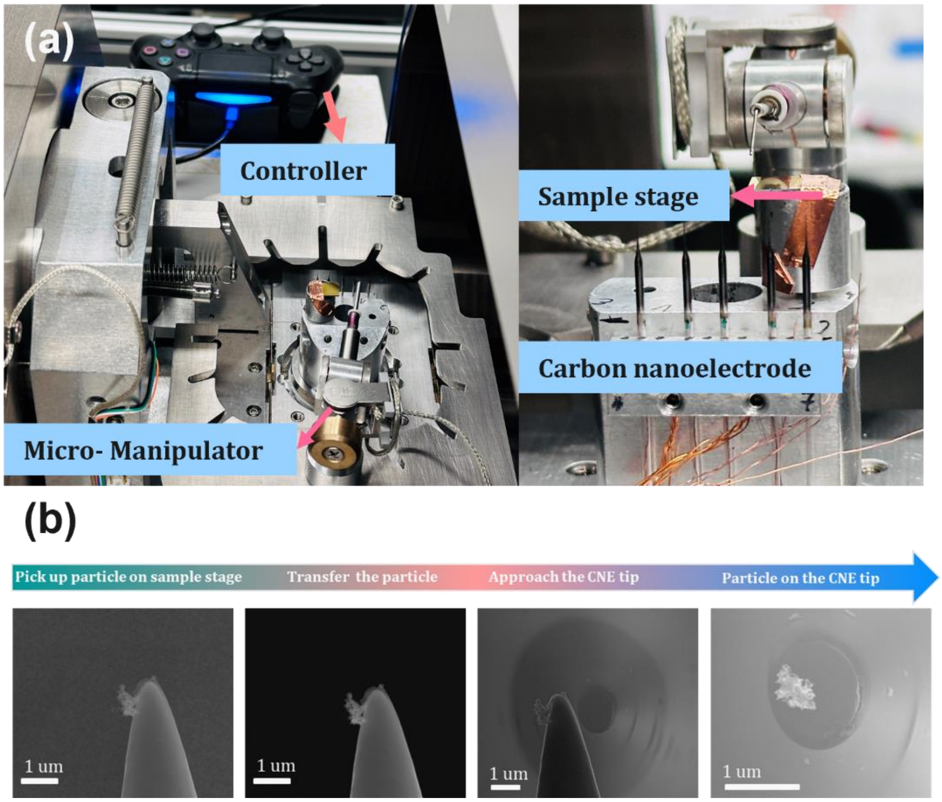

Supplementary Fig. S30 | Process for picking up, transferring, and placing single particle on carbon nanoelectrode. (**a**) Photograph of the micromanipulator set-up installed in the SEM chamber. (**b**) SEM images of details steps of placing single particle on carbon nanoelectrode.

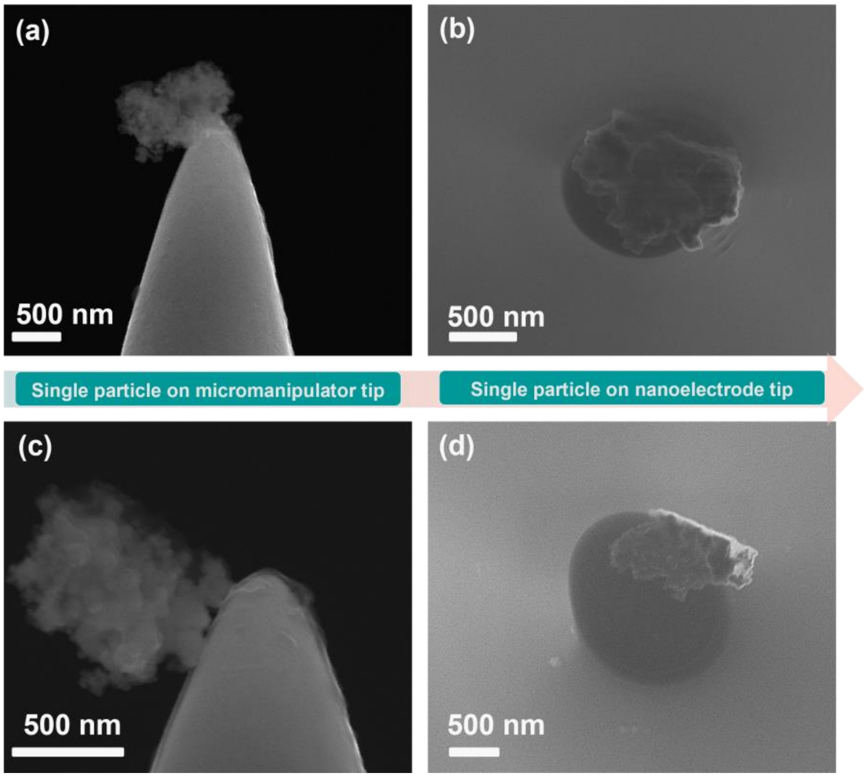

Supplementary Fig. S31 | Micromanipulator inside the SEM with attached single particle and the single particle on the CNE. SEM images of a single paricle of Co-B/Ru_{12} attached to the micromanipulator tip (**a,c**) and single Co-B/Ru_{12} particle placed on a carbon nanoelectrode (**b,d**).

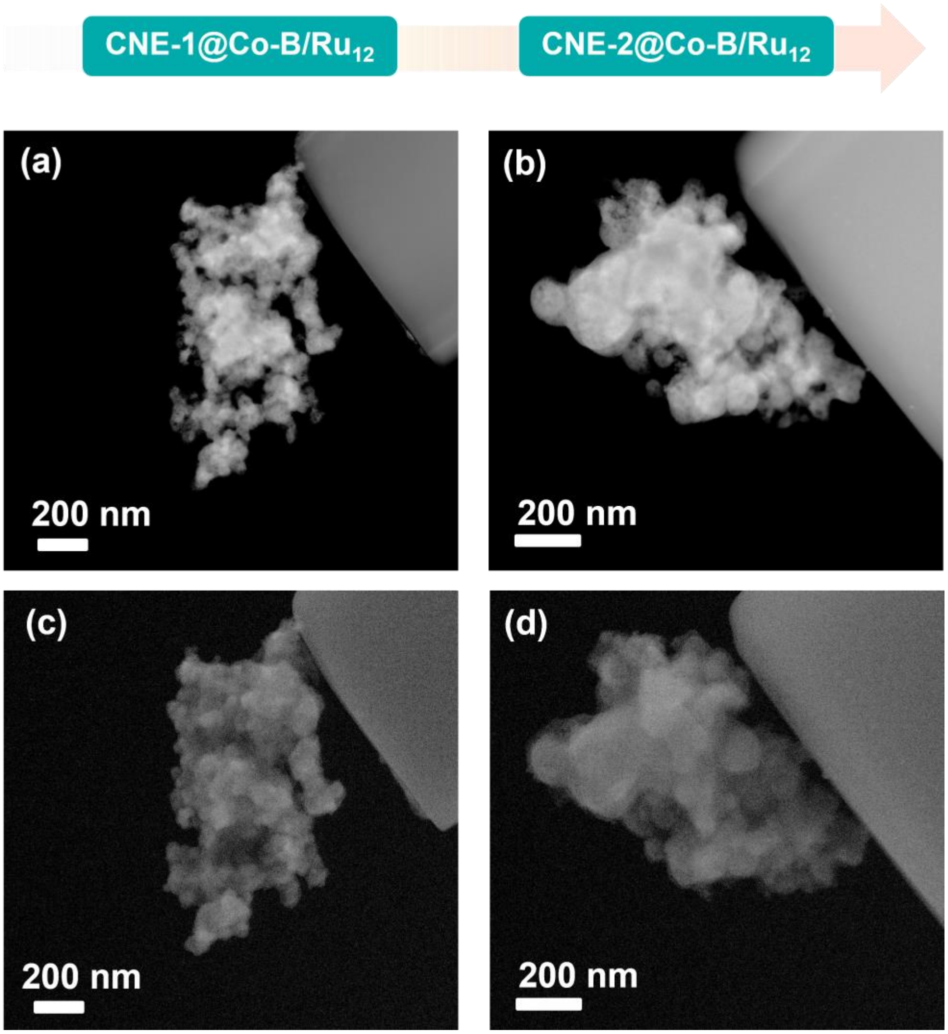

Supplementary Fig. S32 | Morphology of single particle nanoelectrode assemblies. STEM images of CNE-1@Co-B/Ru_{12} (**a**) and CNE-2@Co-B/Ru_{12} (**b**) before CVs. SEM images of CNE-1@Co-B/Ru_{12} (**c**) and CNE-2@Co-B/Ru_{12} (**d**) after CVs.

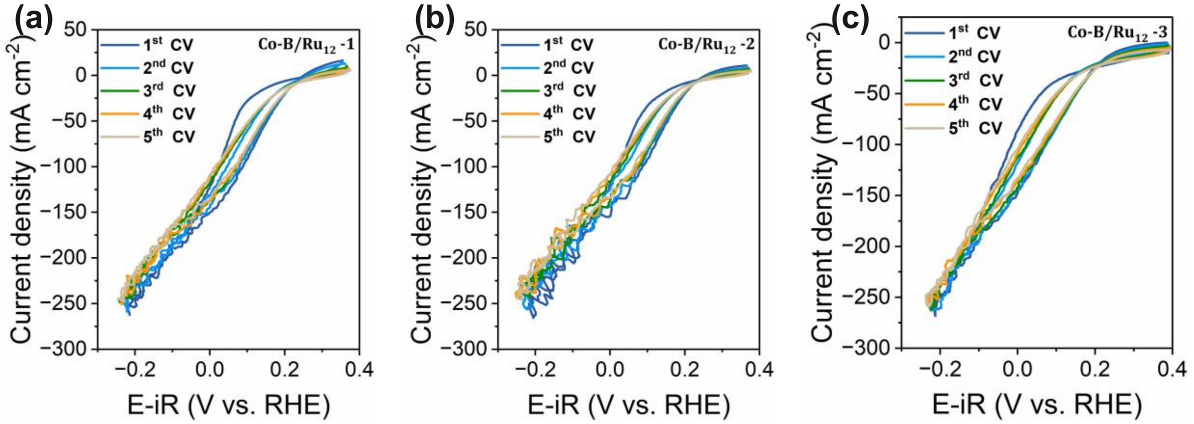

Supplementary Fig. S33 | CV cycles on catalysts-modified carbon paper. Five CV cycles for three independent electrodes, Co-B/Ru_{12}-1 (**a**), Co-B/Ru_{12}-2 (**b**), Co-B/Ru_{12}-3 (**c**), in 0.1 mol L^{-1} NaOH and 0.1 mol L^{-1} $NaNO_3$ with a scan rate of 50 mV s^{-1}.

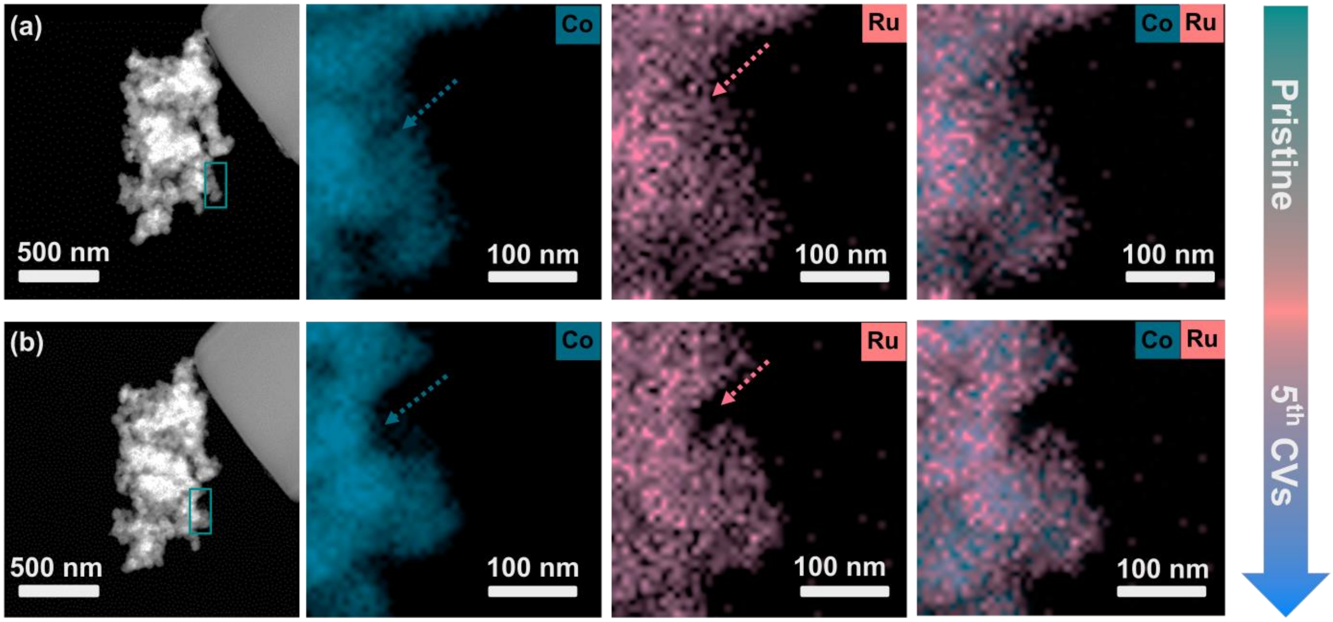

Supplementary Fig. S34 | Structure changes of CNE-1@Co-B/Ru_{12} after the electrocatalytic reaction. STEM images of the fabricated CNE-1@Co-B/Ru_{12} before (**a**) and after 5 CV cycles (**b**) and the corresponding EDS mapping images before (top) and after 5 CV cycles (bottom).

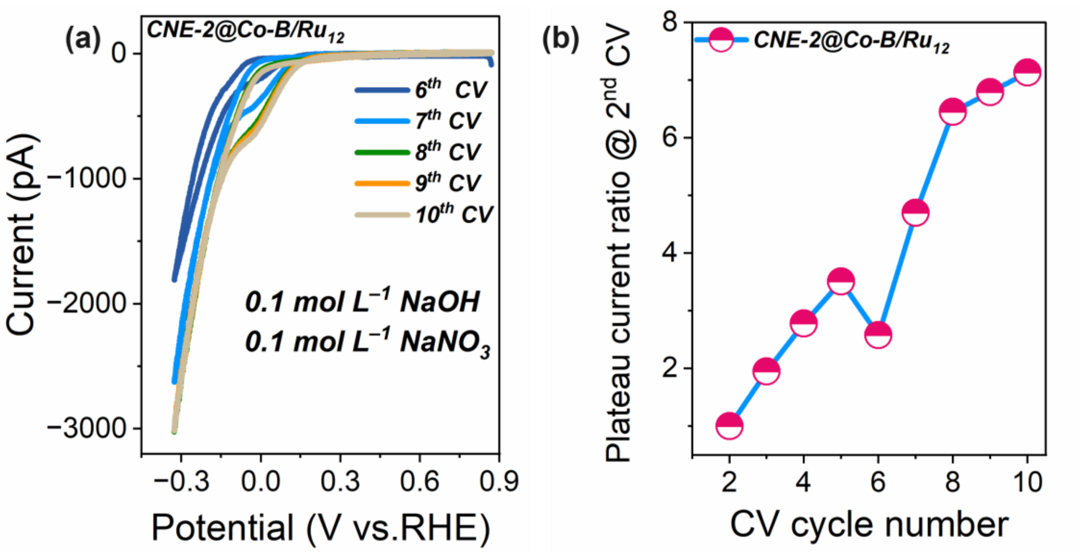

Supplementary Fig. S35 | Activity change trend of CNE-2@Co-B/Ru_{12}. (**a**) 6th to 10th CV cycle of CNE-2@Co-B/Ru_{12} in 0.1 mol L^{-1} NaOH containing the 0.1 mol L^{-1} $NaNO_3$. (**b**) Plateau current ratio of 10th CV scans with respect to the 2nd CV scan of CNE-2@Co-B/Ru_{12}. Potential is not iR-corrected

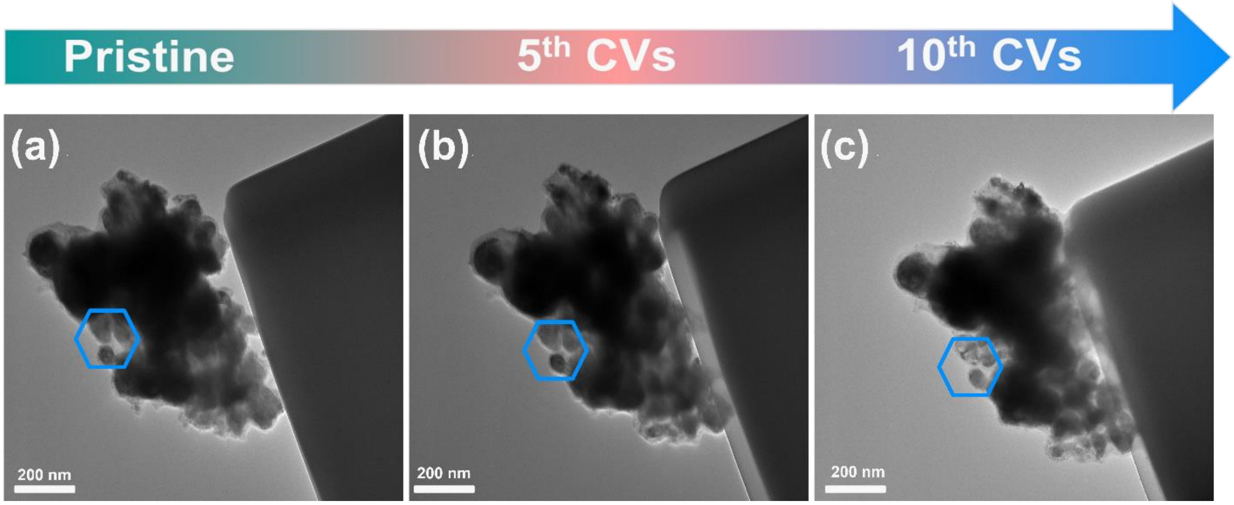

Supplementary Fig. S36 | Structural evolution of CNE-2@Co-B/Ru_{12} after different CV cycles. TEM images of CNE-2@Co-B/Ru_{12} before (**a**), after the 5th CV cycle (**b**), and after the 10th CV cycle (**c**).

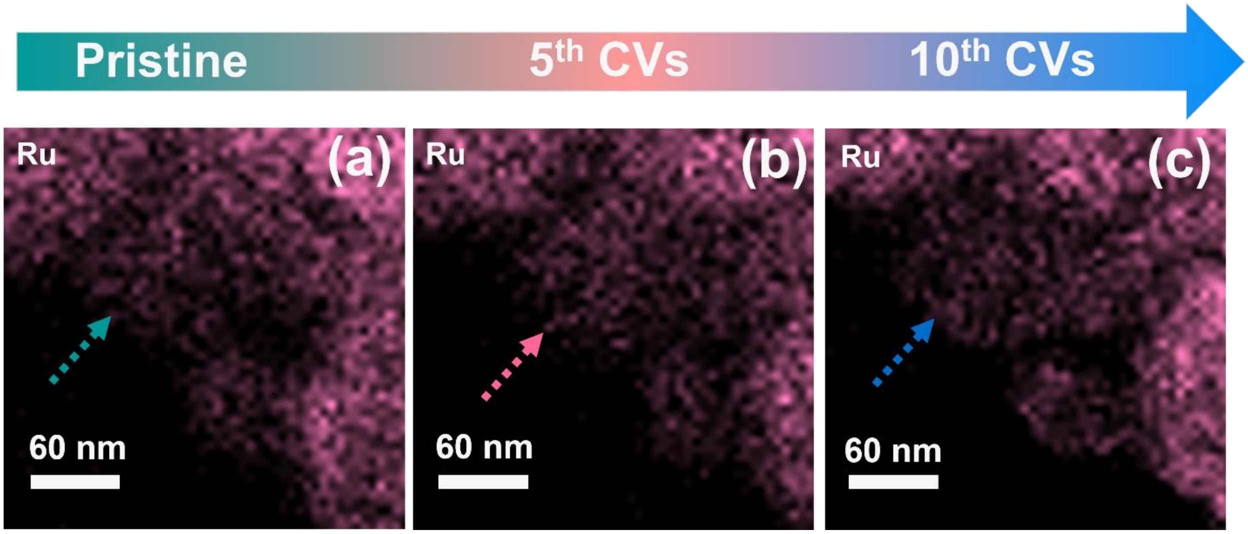

Supplementary Fig. S37 | Ru evolution of CNE-2@Co-B/Ru_{12} at different CV cycles. EDS mapping images of Ru of CNE-2@Co-B/Ru_{12} before (**a**), after the 5th CV cycle (**b**), and after the 10th CV cycle (**c**).

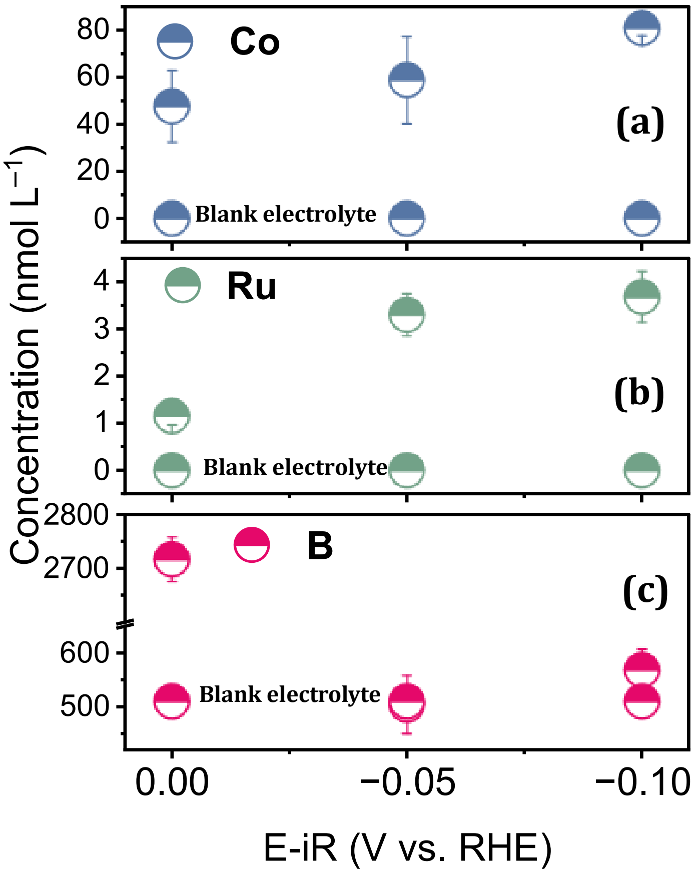

Supplementary Fig. S38 | ICP-MS determined elements concentration in 1-hour post-electrolyzed electrolyte in 0.5 mol L^{-1} $NaNO_3$ at different potentials on Co-B/Ru_{12} modified carbon paper. Concentrations of Co (a), Ru (b), and B (c) at different potentials. After 1 hour of electrolysis at 0 V (vs. RHE), all the post-electrolyzed electrolyte was collected, and the electrochemical cell was cleaned with deionized water before adding fresh electrolyte for the next potential (–0.05 V vs. RHE) electrolysis. The same collection and cleaning procedures were conducted before performing electrolysis at –0.1 V (vs. RHE). The same working electrode was used throughout the 3-stage electrolysis. Error bars denote the standard deviations from three independent measurements.

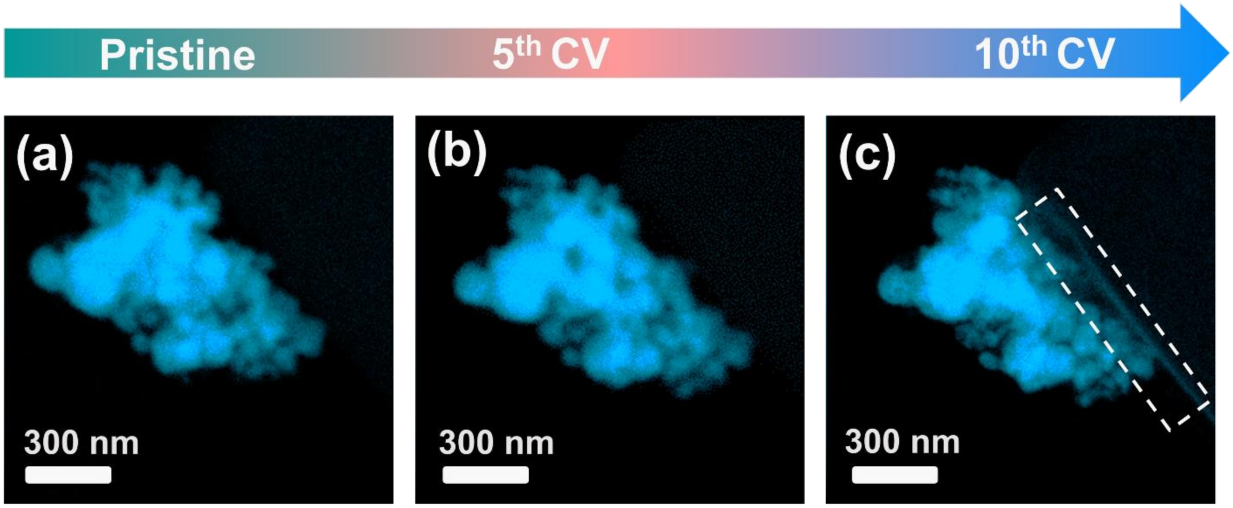

Supplementary Fig. S39 | Ru evolution of CNE-2@Co-B/Ru_{12} at different CV cycles. EDS mapping images of Co of CNE-2@Co-B/Ru_{12} before (**a**), after the 5th CV cycle (**b**), and after the 10th CV cycle (**c**).

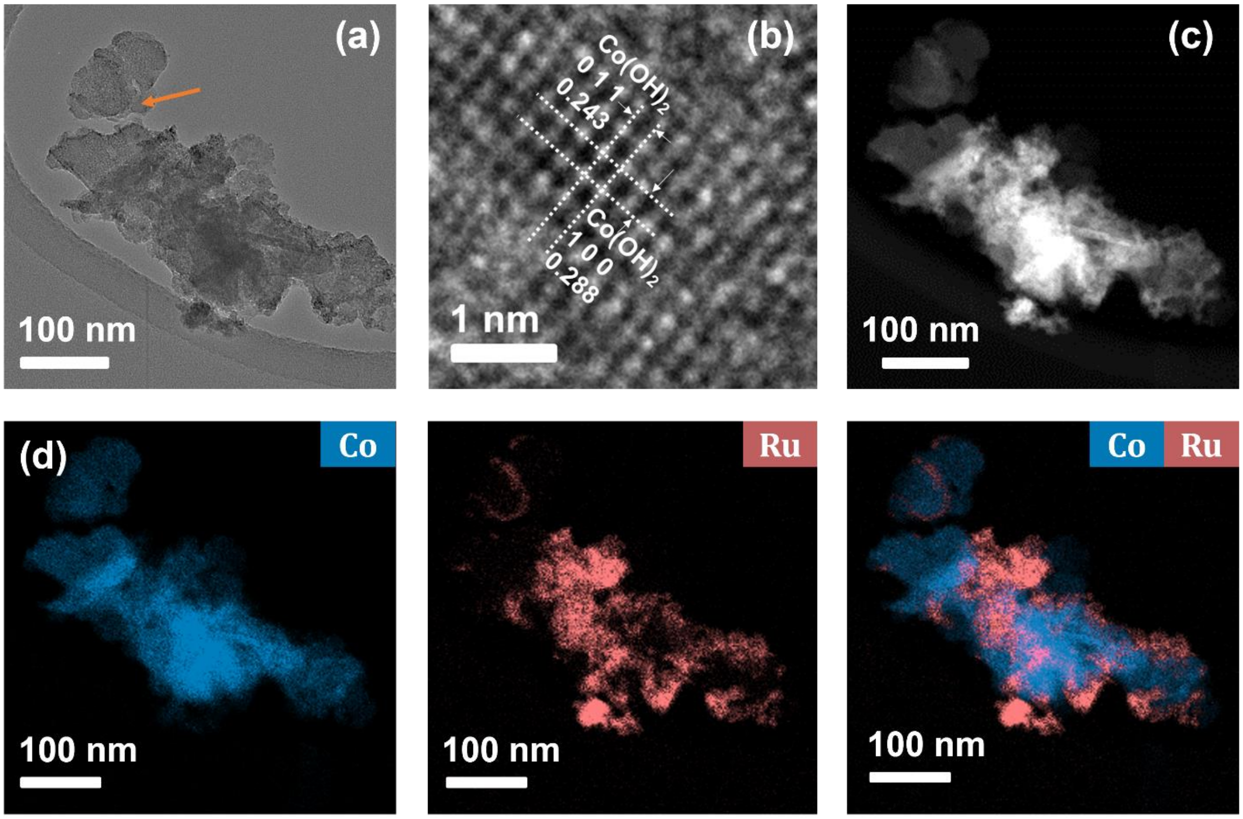

Supplementary Fig. S40 | Structure of Co-B/Ru_{12} after reaction in 0.1 mol L^{-1} $NaNO_3$. (a) TEM image of Co-B/Ru_{12} after 10 hours chronoamperometry at –0.1 V (vs. RHE). (**b**) HRTEM image of Co-B/Ru_{12} after 10 hours chronoamperometry at –0.1 V (vs. RHE). (**c**) STEM image of Co-B/Ru_{12} after 10 hours chronoamperometry at –0.1 V (vs. RHE). (**d**) EDX mapping images of Co-B/Ru_{12} after 10 hours chronoamperometry at –0.1 V (vs. RHE).

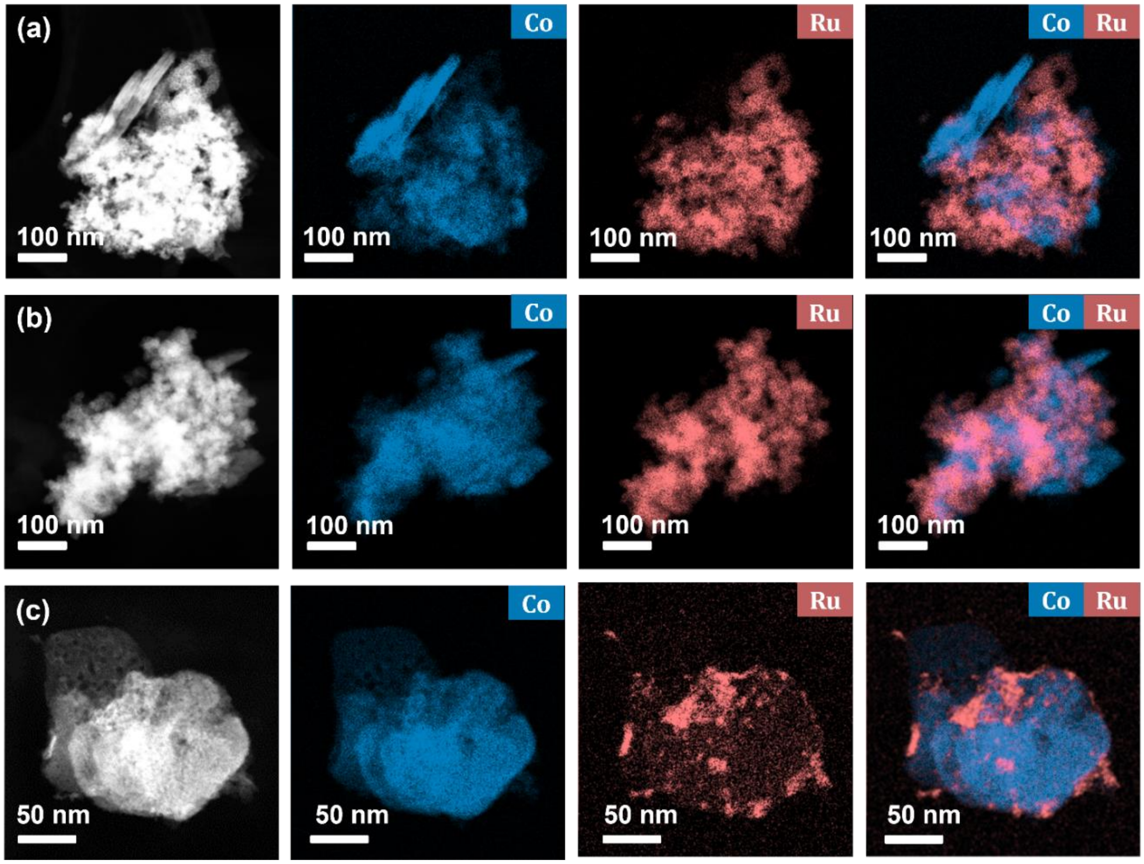

Supplementary Fig. S41 | Structure of Co-B/Ru$_{12}$ after reaction in 0.5 mol L^{-1} $NaNO_3$**. (a-c)** three **S**TEM images and corresponding EDS mapping images of Co-B/Ru$_{12}$ after 30 hours chronoamperometry at –0.1 V (vs. RHE).

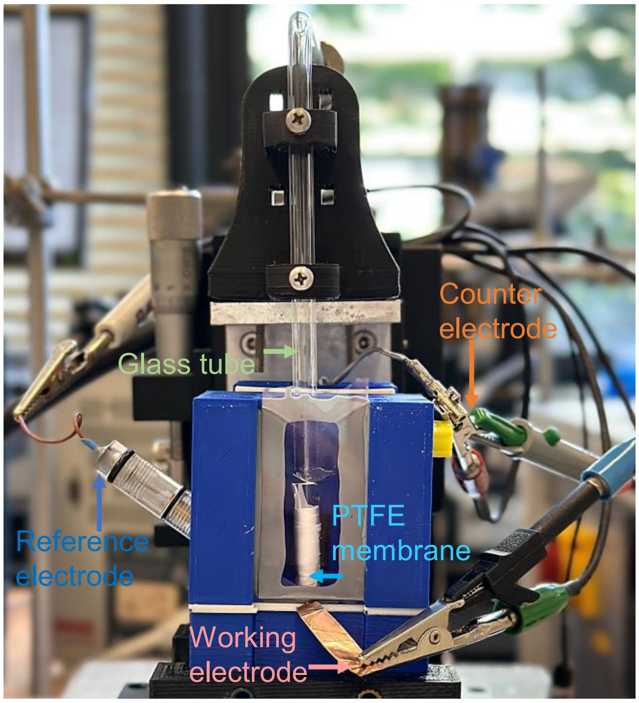

Supplementary Fig. S42 | Electrochemical Cell for DEMS. Photograph of 3D-printed electrothermal cell with a custom-made mass-spectrometry (MS) tip.

Supplementary Table S1 | Comparison of NH_3 synthesis activity of Co-B/Ru_{12} with other catalysts for NO_3RR at ambient conditions.

Catalysts	Electrolyte	Potential (V vs. RHE)	NH_3 yield rate	FE_{NH3} (%)	Double layer capacitance	Reference
Co-B/Ru_{12} on carbon paper	0.1 mol L^{-1} NaOH and 0.1 mol L^{-1} $NaNO_3$	0.0V	7.4 ± 0.6 mg h^{-1} cm^{-2}	90.4 ± 9.2%	1.16 mF cm^{-2}	This work
Co-B/Ru_{12} on carbon paper	0.1 mol L^{-1} NaOH and 0.1 mol L^{-1} $NaNO_3$	–0.2 V	15.0 ± 0.7 mg h^{-1} cm^{-2}	87.5± 4.8%	1.16 mF cm^{-2}	This work
Co-B/Ru_{12} on carbon paper	0.1 mol L^{-1} NaOH and 0.5mol L^{-1} $NaNO_3$	–0.1 V	47.6± 2.7 mg h^{-1} cm^{-2}	83.7± 3.4%	1.16 mF cm^{-2}	This work
Pd/TiO_2 on carbon cloth	1 mol L^{-1} LiCl and 0.25 mol $L^{-1}NO_3^-$	−0.7V	1.12 mg h^{-1} cm^{-2}	92.1%	5.51 mF cm^{-2}	Energy Environ. Sci. 2021, 14, 3938–3944.[1]
Cu_2O+Co_3O_4 on carbon paper	0.1 mol L^{-1} NaOH and 0.1 mol L^{-1} $NaNO_3$	−0.3V	12.76 mg h^{-1} cm^{-2}	85.4%	0.84 mF cm^{-2}	Angew. Chem. Int. Ed. 2023, 62, e202214830[2]
PdCu nanocube on carbon paper	1 mol L^{-1} KOH and 1 mol $L^{-1}NO_3^-$	−0.2V	≈1.7 mg h^{-1} cm^{-2}	≈72 %	3.59 mF cm^{-2}	Nat. Commun. 2022, 13, 2338[3]
Cu/CuAu ordered SAA on carbon paper	1 mol L^{-1} KOH and 1 mol $L^{-1}NO_3^-$	−0.2V	≈2.72 mg h^{-1} cm^{-2}	≈62 %	4.91 mF cm^{-2}	Nat. Synth. 2023, 2, 624[4]
Rh@Cu 0.6% on Cu foil	0.1 mol L^{-1} KOH and 0.1 mol $L^{-1}NO_3^-$	−0.2V	≈13.5 mg h^{-1} cm^{-2}	93%	N/A	Angew. Chem. Int. Ed. 2022,134, e202202556[5]
Co_3CuN on carbon paper	0.5 mol L^{-1} KOH and 0.032 mol $L^{-1}NO_3^-$	−0.3V	7.74 mg h^{-1} cm^{-2}	97%	N/A	Angew. Chem. Int. Ed. 2023, 62, e202308775[6]
Ni_{35}/NC-sd on Ti mesh	0.5 mol L^{-1} Na_2SO_4 and 0.3 mol $L^{-1}NO_3^-$	−0.5V	1.2 mg h^{-1} cm^{-2}	99%	N/A	Angew. Chem. Int. Ed. 2021, 60, 20711–20716[7]
Fe/Ni_2P on carbon cloth	0.2 mol L^{-1} K_2SO_4 and 0.05 mol $L^{-1}NO_3^-$	−0.4V	4.16 mg h^{-1} cm^{-2}	94.3%	N/A	Adv. Energy Mater. 2022, 12, 2103872[8]
Fe SAC on glassy carbon	0.1 mol L^{-1} K_2SO_4 and 0.5 mol $L^{-1}NO_3^-$	−0.66V	≈1.95 mg h^{-1} cm^{-2}	75%	N/A	Nat. Commun. 2021, 12, 2870[9]
Strained Ru nanoclusters on carbon paper	1 mol L^{-1} KOH and 1 mol $L^{-1}NO_3^-$	−0.2V	19.89 mg h^{-1} cm^{-2}	≈100%	0.48 mF cm^{-2}	J. Am. Chem. Soc. 2020, 142, 7036–7046[10]
Meso-PdN NCs on carbon paper	0.1 mol L^{-1} Na_2SO_4 and 0.005mol $L^{-1}NO_3^-$	-0.7V	0.376 mg h^{-1} cm^{-2}	96%	1.17 mF cm^{-2}	Adv. Mater.2023, 35, 2207305[11]
Ru/Cu_2O on Cu foam	1 mol L^{-1} KOH and 1 mol $L^{-1}NO_3^-$	-0.4 V	119 mg h^{-1} cm^{-2}	75%	449 mF cm^{-2}	J. Am. Chem. Soc. 2024, 146, 668–676[12]
Ru/CuNW on Cu foam	1 mol L^{-1} KOH and 0.032 mol $L^{-1}NO_3^-$	-0.135 V	76.5 mg h^{-1} cm^{-2}	96%	740.6 mF cm^{-2}	Nat. Nanotechnol.2022, 17, 757-769[13]
$Pd_{74}Ru_{26}$ on carbon fiber paper	1 mol L^{-1} KOH and 0.032 mol $L^{-1}NO_3^-$	–0.5 V	20.6 mg h^{-1} cm^{-2}	91.6%	110.8 mF cm^{-2}	Chem. Sci., 2024, 15, 8204–8215[14]
Ru-Fe_2O_3 on carbon cloth	0.5 mol L^{-1} Na_2SO_4 and 0. 1 mol $L^{-1}NO_3^-$	-0.9 V	5.6 mg h^{-1} cm^{-2}	72:8%	4.74 mF cm^{-2}	Appl. Catal., B,2024, 351,123967[15]
Ru SASs/Co HNSs on carbon paper	1 mol L^{-1} NaOH and 1 mol L^{-1} $NaNO_3$	–0.3V	8.211 mg h^{-1} cm^{-2}	100%	N/A	Chem. Eng. J.; 2024,490, 151883[16]
Ru SA-NC on carbon paper	1 mol L^{-1} KOH and 0.5 mol L^{-1} KNO_3	-0.6 V	2.278 mg h^{-1} cm^{-2}	72.8%	N/A	ACS Nano, 2023, 17, 3483-3491[17]
RuOx/Pd on carbon cloth	1 mol $^{L-1}$ KOH and 0.51mol L^{-1} KNO_3	-0.5 V	23.5 mg h^{-1} cm^{-2}	98.6%	N/A	ACS Nano, 2023, 17, 1081-1090[18]

Supplementary Table S2 | Comparison of the ammonia synthesis rate of our Co-B/Ru12 electrocatalyzed NO3RR with those of Haber-Bosch process at lab scale (milder reaction conditions) and of the state-of-the-art electrocatalytic and photocatalytic NRR routes

Ammonia synthesis route	Catalyst	NH_3 synthesis rate	Operation condition	Reference
Haber-Bosch process	Ba_2RuH_6/MgO	35 mmol $g^{-1}_{cat}\,h^{-1}$	300°C and 10 bar	Nat. Catal. 2021,11,959-967[19]
Haber-Bosch process	Ni/LaN	5.543 mmol $g^{-1}_{cat}\,h^{-1}$	400°C and 1 bar	Nature 2020,7816,391-395[20]
Haber Bosch process	Ru/LaCoSi	18.5 mmol $g^{-1}_{cat}\,h^{-1}$	400°C and 9 bar	J. Am. Chem. Soc.2022,144,8683-8692[21]
N_2 reduction reaction	Ru single atom catalysts	7.12 mmol $g^{-1}_{cat}\,h^{-1}$	25°C; ambient pressure	Adv. Mater. 2018, 30, 1803498[22]
N_2 reduction reaction	Proton-filtering covalent organic frameworks	16.89 mmol $g^{-1}_{cat}\,h^{-1}$	25°C; ambient pressure	Nat. Catal. 2021,4, 322-331[23]
NO_3^- reduction reaction	$Co\text{-}B/Ru_{12}$	5597 mmol $g^{-1}_{cat}\,h^{-1}$	25°C; ambient pressure	This work

Supplementary references

1. Guo, Y. *et al.* Pd doping-weakened intermediate adsorption to promote electrocatalytic nitrate reduction on TiO_2 nanoarrays for ammonia production and energy supply with zinc–nitrate batteries. *Energy Environ. Sci.* **14,** 3938–3944 (2021).
2. Zhang, J. *et al.* Single-entity Electrochemistry Unveils Dynamic Transformation during Tandem Catalysis of Cu_2O and Co_3O_4 for Converting NO_3^- to NH_3. *Angew. Chem., Int. Ed.,* e202214830 (2023).
3. Gao, Q. *et al.* Breaking adsorption-energy scaling limitations of electrocatalytic nitrate reduction on intermetallic CuPd nanocubes by machine-learned insights. *Nat. Commun.* **13,** 2338 (2022).
4. Gao, Q. *et al.* Synthesis of core/shell nanocrystals with ordered intermetallic single-atom alloy layers for nitrate electroreduction to ammonia. *Nat. Synth* **2,** 624–634 (2023).
5. Liu, H. *et al.* Efficient Electrochemical Nitrate Reduction to Ammonia with Copper-Supported Rhodium Cluster and Single-Atom Catalysts. *Angew. Chem., Int. Ed.* **61,** e202202556 (2022).
6. Gong, Z. *et al.* Modulating Metal-Nitrogen Coupling in Anti-Perovskite Nitride via Cation Doping for Efficient Reduction of Nitrate to Ammonia. *Angew. Chem., Int. Ed.* **62,** e202308775 (2023).
7. Gao, P. *et al.* Schottky barrier-induced surface electric field boosts universal reduction of NO_x^- in water to ammonia. *Angew. Chem., Int. Ed.*, 20711–20716 (2021).
8. Zhang, R. *et al.* Efficient Ammonia Electrosynthesis and Energy Conversion through a Zn-Nitrate Battery by Iron Doping Engineered Nickel Phosphide Catalyst. *Adv. Energy Mater.* **12,** 2103872 (2022).
9. Wu, Z.-Y. *et al.* Electrochemical ammonia synthesis via nitrate reduction on Fe single atom catalyst. *Nat. Commun.* **12,** 2870 (2021).
10. Li, J. *et al.* Efficient Ammonia Electrosynthesis from Nitrate on Strained Ruthenium Nanoclusters. *J. Am. Chem. Soc.* **142,** 7036–7046 (2020).
11. Sun, L. & Liu, B. Mesoporous PdN Alloy Nanocubes for Efficient Electrochemical Nitrate Reduction to Ammonia. *Adv. Mater.* **35,** e2207305 (2023).
12. Hu, Q. *et al.* Ammonia Electrosynthesis from Nitrate Using a Ruthenium-Copper Cocatalyst System: A Full Concentration Range Study. *J. Am. Chem. Soc.* **146,** 668–676 (2024).
13. Chen, F.-Y. *et al.* Efficient conversion of low-concentration nitrate sources into ammonia on a Ru-dispersed Cu nanowire electrocatalyst. *Nat. Nanotechnol.,* 757–769 (2022).
14. Hu, Y. *et al.* Alloying Pd with Ru enables electroreduction of nitrate to ammonia with ~100% faradaic efficiency over a wide potential window. *Chem. Sci.* **15,** 8204–8215 (2024).
15. Luo, S. *et al.* Ruthenium-induced hydrolysis effect on Fe_2O_3 nanoarrays for high-performance electrochemical nitrate reduction to ammonia. *Appl. Catal., B* **351,** 123967 (2024).
16. Cao, H. *et al.* Constructing Ru single-atomic sites through potential-induced self-reconstruction to accelerate electrocatalytic nitrate reduction for ammonia production. *Chem. Eng. J.* **490,** 151883 (2024).
17. Ke, Z. *et al.* Selective NO_x^- Electroreduction to Ammonia on Isolated Ru Sites. *ACS nano* **17,** 3483–3491 (2023).
18. Li, X., Shen, P., Li, X., Ma, D. & Chu, K. Sub-nm RuOx Clusters on Pd Metallene for Synergistically Enhanced Nitrate Electroreduction to Ammonia. *ACS nano* **17,** 1081–1090 (2023).
19. Wang, Q. *et al.* Ternary ruthenium complex hydrides for ammonia synthesis via the associative mechanism. *Nat Catal* **4,** 959–967 (2021).
20. Ye, T.-N. *et al.* Vacancy-enabled N_2 activation for ammonia synthesis on an Ni-loaded catalyst. *Nature* **583,** 391–395 (2020).
21. Gong, Y. *et al.* Unique Catalytic Mechanism for Ru-Loaded Ternary Intermetallic Electrides for Ammonia Synthesis. *J. Am. Chem. Soc.* **144,** 8683–8692 (2022).
22. Geng, Z. *et al.* Achieving a Record-High Yield Rate of 120.9 μgNH3 mgcat.-1 h-1 for N2 Electrochemical Reduction over Ru Single-Atom Catalysts. *Adv. Mater.,* e1803498 (2018).
23. Liu, S. *et al.* Proton-filtering covalent organic frameworks with superior nitrogen penetration flux promote ambient ammonia synthesis. *Nat Catal* **4,** 322–331 (2021).
